# Controlling the Processability and Stability of Supramolecular Polymers Using the Interplay of Intra- and Intermolecular Interactions

**DOI:** 10.1021/acs.macromol.2c00976

**Published:** 2022-07-27

**Authors:** Joost J. B. van der Tol, Ghislaine Vantomme, Anja R. A. Palmans, E. W. Meijer

**Affiliations:** Institute for Complex Molecular Systems and Laboratory of Macromolecular and Organic Chemistry, Eindhoven University of Technology, P.O. Box 513, 5600 MB Eindhoven, The Netherlands

## Abstract

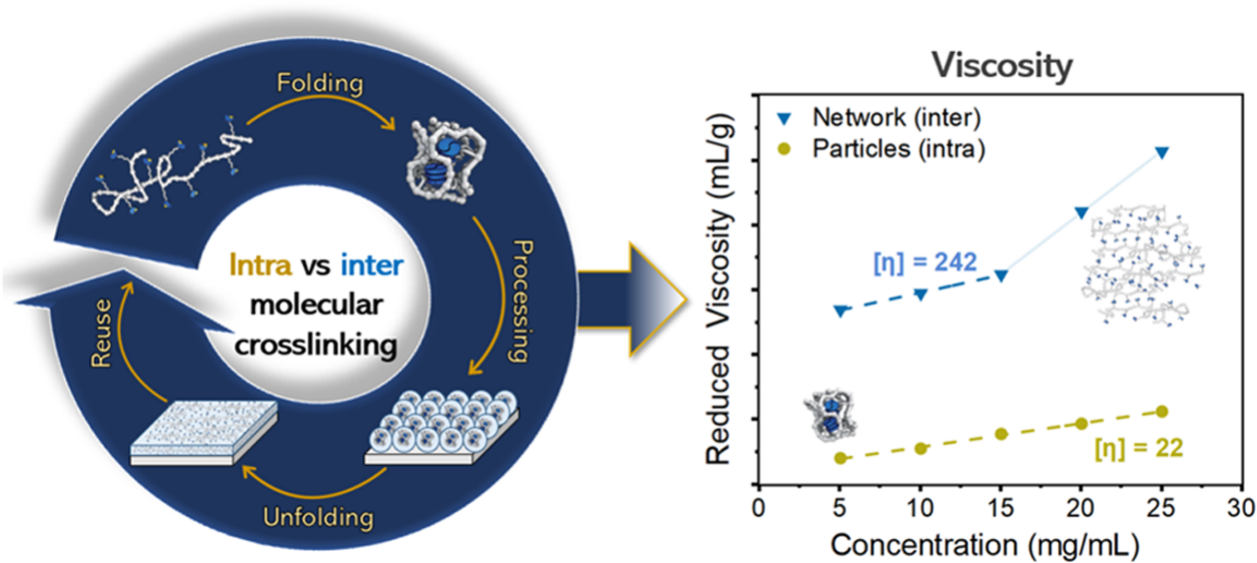

Polymer networks crosslinked via non-covalent interactions afford interesting materials for a wide range of applications due to their self-healing capability, recyclability, and tunable material properties. However, when strong non-covalent binding motifs in combination with high crosslink density are used, processing of the materials becomes troublesome because of high viscosities and the formation of insoluble gels. Here, we present an approach to control the processability of grafted polymers containing strong non-covalent interactions by balancing the interplay of intra- and intermolecular hydrogen bonding. A library of copolymers with different degrees of polymerization and content of protected ureido-pyrimidinone-urea (UPy-urea) grafts was prepared. Photo-deprotection in a good solvent like tetrahydrofuran (THF) at low concentrations (≤1 mg mL^–1^) created intramolecularly assembled nanoparticles. Remarkably, the intrinsic viscosity of these nanoparticle solutions was an order of magnitude lower compared to solutions of the intermolecularly assembled analogues, highlighting the crucial role of intra- versus intermolecular interactions. Due to the strong hydrogen bonds between UPy dimers, the intramolecularly assembled structures were kinetically trapped. As a result, the polymer nanoparticles were readily processed into a bulk material, without causing major changes in the morphology as verified by atomic force microscopy. Subsequent intermolecular crosslinking of the nanoparticle film, by heating to temperatures where the hydrogen-bond exchange becomes fast, resulted in a crosslinked network. The reversibility of the hereby obtained polymer networks was shown by retrieving the intramolecularly assembled nanoparticles via redissolution and sonication of the intermolecularly crosslinked film in THF with a small amount of acid. Our results highlight that the stability and processability of highly supramolecularly crosslinked polymers can be controlled both in solution and in bulk by using the interplay of intra- and intermolecular non-covalent interactions in grafted polymers.

## Introduction

In our current polymer-infused society, huge quantities of polymers are produced everyday and many of these end as waste, contributing to environmental pollution.^1^ This has urged scientists to develop sustainable “dynamic polymer systems”^2^ such as dynamic covalent^3−7^ and non-covalent polymers.^8−12^ The incorporated dynamicity enables polymers to be reprocessed multiple times in almost any shape via thermodynamic control.^13^ In dynamic covalent polymers, labile covalent bonds allow for reprocessing via dissociative and associative mechanisms, giving the polymer the ability to be reshaped.^14^ However, high temperatures and pressures are often required in order to induce sufficient mobility and to trigger the bond exchange.^15−17^ On the contrary, non-covalent polymers generally relax at shorter time scales (minutes compared to hours), enabling faster dynamics even at room temperature.^18,19^ In addition, the rate of association and dissociation of non-covalent interactions is highly dependent on temperature. This provides non-covalent polymers with excellent processing properties as shown by linear chain extension of low molecular weight polymers with hydrogen bonding motifs.^20,21^ Using a strong ureidopyrimidinone (UPy) motif, our group achieved material properties similar to conventional macromolecules at room temperature yet exhibiting superb processing properties at slightly elevated temperatures.^22^

Besides telechelic non-covalent polymers, various architectures and non-covalent motifs have been developed over the past decades, giving rise to a wide range of precisely tailorable material properties.^23−27^ However, processing becomes more troublesome when multifunctional polymers containing strong non-covalent bonds are used to achieve high performance functional, self-healing, and adaptable materials.^28^ Due to the high degree of connectivity, polymers exhibit extremely long relaxation times (up to days), leading to high viscosities or the formation of insoluble gels during processing.^29^ Besides supramolecular interactions, entanglements play a crucial role in processing as well since they significantly slow down the dynamics of the polymer.^30^ Logically, an effective approach to circumvent this problem is to restrain the polymer chains from forming entanglements, which was nicely shown by Kuhn and Balmer^31^ and later by Hawker et al.^32^ using covalently crosslinked single-chain polymeric nanoparticles. They prepared a copolymer solution containing isocyanate functionalities after which various diamines were added under dilute conditions to intramolecularly crosslink the polymers. Subsequent viscosity studies demonstrated the strength of this approach by achieving significantly lower viscosities for the nanoparticles compared to the random coil analogues. At the same time, our group showed the facile preparation of metastable polymeric nanoparticles based on hydrogen bonding potentially applicable for solution processable supramolecular materials.^33,34^ This concept has been used to study single-chain polymer nanoparticles (SCPNs);^35,36^ however, this has not been worked out with the idea to broaden the scope of processing supramolecular polymers into widely applied sustainable materials.

**Figure 1:**
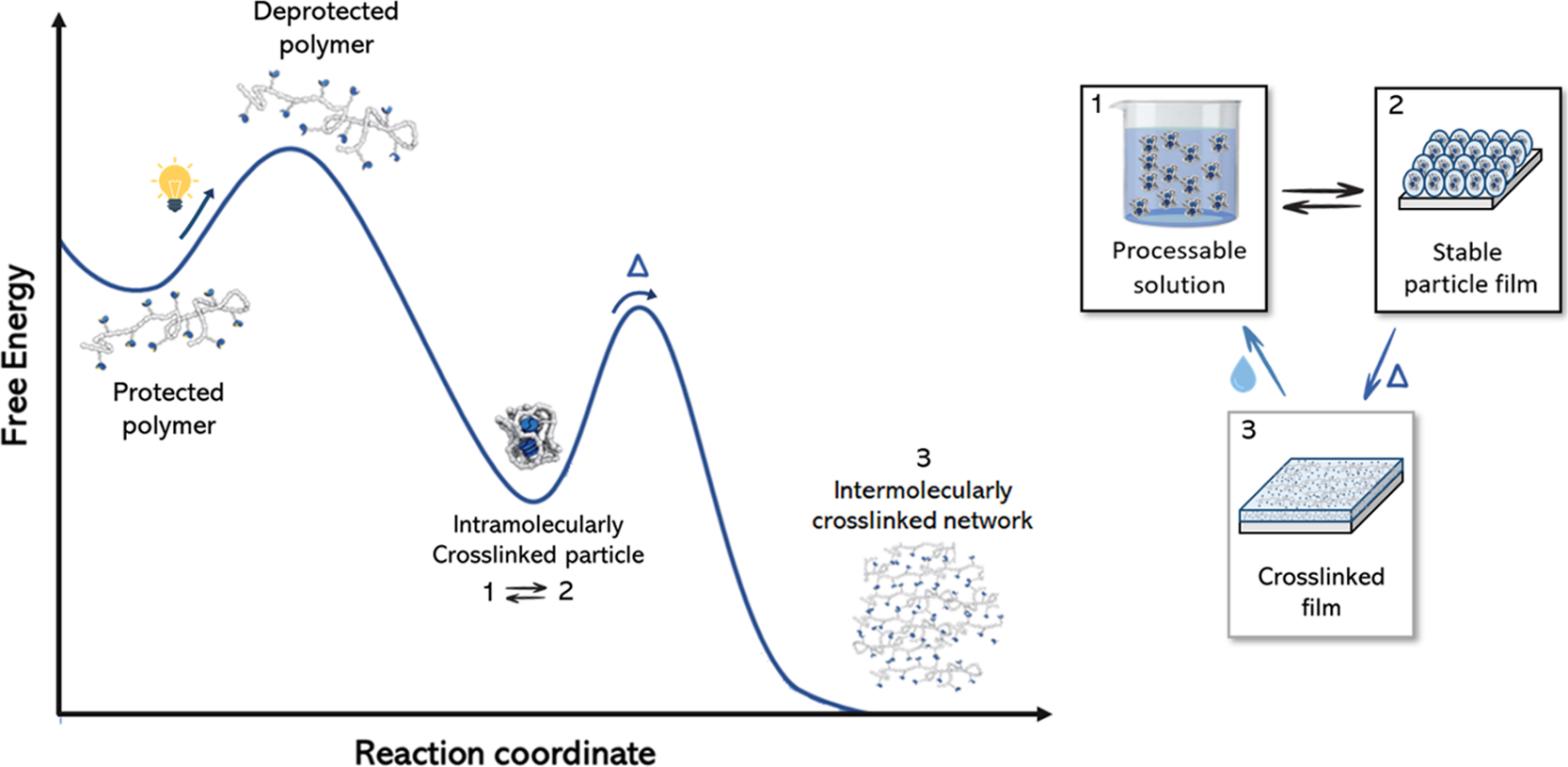
Schematic representation of the energy diagram for sequential intramolecular assembly and intra-to-intermolecular assembly of UPy-urea grafted polymers into intramolecularly crosslinked nanoparticles (1) and an intermolecularly crosslinked network (3), respectively. In the intermediate kinetically trapped state, the intramolecularly crosslinked nanoparticles exhibit significantly lower viscosities allowing for easier processing into stable particle films (2).

Herein, we report a strategy to enhance the processability and to control the stability of supramolecular grafted polymers by controlling intra- versus intermolecular interactions. In this study, ring-opening metathesis polymerization (ROMP) of norbornene monomers using a second generation Grubbs catalyst is applied due to easy functionalization and high resilience of the catalyst toward supramolecular moieties that can be obtained in high quantities. A library of norbornene polymers, grafted with protected UPy supramolecular motifs, is synthesized followed by intramolecular assembly into polymer nanoparticles in a controlled fashion by selective photo-deprotection using 365 nm light. Herein, the strongly dimerizing UPy groups complemented with lateral stacking of urea moieties^37,38^ and high glass transition temperatures (*T*_g_) of polynorbornene ensured the formation of stable, intramolecularly crosslinked nanoparticles. Weak hydrogen bonding motifs,^39−42^ in contrast, exhibit fast exchange dynamics, resulting in higher probabilities of intermolecular crosslinking, and are therefore less suitable for this processing approach.^35^ The solvent generally plays a crucial role in this process as strong hydrogen bonding complexes should be formed while at the same time interparticle aggregation should be prevented. Previous studies on similar UPy grafted polymer systems showed that tetrahydrofuran (THF) works well^43^ and will therefore be predominantly used as a solvent. After intramolecular assembly, the particle processing (Figure 1, 1 → 2) and the intra-to-intermolecular rearrangement (Figure 1, 2 → 3) are studied. This work provides structural insights into the relation between intra- and intermolecular non-covalent interactions and reveals how this interplay can be used to regulate the solution viscosity and stability of supramolecular grafted polymers during processing. Moreover, polymer redissolution studies are presented as a proof of concept to address the reversibility of the system (Figure 1, 3 → 1 and 2 → 1) and therewith the potential of this sustainable and novel processing approach toward high-performance supramolecular materials.

## Results and Discussion

### Synthetic Design and Synthesis

The synthetic approach of the monomers and polymers is similar to that described in previous work^44^ from our group (a complete reaction scheme can be seen in the Supporting Information). Here, we designed and synthesized a library of poly(norbornenes) grafted with hydrogen bonding building blocks using ring-opening metathesis polymerization (ROMP). The *N*-substituted *cis*-5-norbornene-2,3-dicarboxylic imides enable straightforward functionalization. Polymerization using the second generation Grubbs catalyst provided high monomer conversions as the first and third generation Grubbs catalysts resulted in significantly lower conversions. In order to intramolecularly assemble the polymers into nanoparticles, we use *o*-nitrobenzyl protected UPy as the precursor of the supramolecular motif with an adjacent urea moiety for lateral stacking.^37,38^ The comonomer is functionalized with an aliphatic dodecyl chain that provides solubility to the polymeric nanoparticles. With the aim of examining various structural effects on the nanoparticle stability, we deliberately prepared a library of polymers containing 0, 5, and 10 mol % UPy-urea as comonomers with varying degrees of polymerization (100, 250, and 500).

First, an amine-functionalized norbornene was acquired by a condensation reaction of endo/exo- *cis*-5-norbornene-2,3-dicarboxylic anhydride and an excess of 1,12-diaminododecane in the presence of triethylamine. Subsequent addition of 1-(6-isocyanatohexyl)-3-(6-methyl-4-oxo-1,4-dihydropyrimidin-2-yl)urea followed by protection of the UPy with *o*-nitrobenzyl chloride without intermediate purification yielded the supramolecular norbornene monomer. The dodecyl-functionalized norbornene monomer was obtained in high yields via a single condensation step of *n-*dodecylamine with the norbornene precursor (Scheme S1).

Using ROMP and the second generation Grubbs catalyst, the majority of (co)polymers (**P1**–**P5**, **P7**, and **P8**) was successfully prepared at room temperature in dichloromethane, achieving high monomer conversions (97–99%, Table 1) and a high degree of incorporation of functional monomer **5** (Scheme S2). Copolymers **P6** and **P9** (high molecular weight copolymers) were prepared at 50 °C in dichloroethane, obtaining slightly lower monomer conversions of 80 and 88%, respectively. A higher temperature was applied for these copolymerizations to prevent the complexation of protected UPy with small amounts of Grubbs catalyst.^45^ The conversion and incorporation of functional protected monomers was analyzed using ^1^H-NMR spectroscopy (Table 1). The full molecular characterization of the copolymers can be found in the SI (Figures S1–S7).

**Table 1:** Characterization of Polymers **P1–P9** by NMR and SEC

polymer^a^	UPy feed^b^ [mol %]	conversion^c^ [%]	observed UPy^d^ [mol %]	DP^e^	*M*_n_^f^ [KDa]	*Đ*^g^
**P1**	0	100		100	51	2.15
**P2**	0	99		247	76	2.16
**P3**	0	99		495	135	2.06
**P4**	5	100	4	100	55	2.05
**P5**	5	99	5	248	84	1.81
**P6**	5	80	5	400	187	1.70
**P7**	10	99	9	99	66	1.85
**P8**	10	97	8	242	89	1.92
**P9**	10	88	8	440	161	1.45

a Polymers as depicted in Figure 2.

b Theoretical molar percentage of UPy-urea monomer **5** incorporated into the polymer.

c Monomer conversion determined by ^1^H-NMR spectroscopy in chloroform-*d*.

d Observed molar percentage of UPy-urea monomer **5** incorporated into the polymer.

e Degree of polymerization (DP) calculated from the monomer conversion.

f Number average molecular weight (*M*_n_) measured by SEC at 1 mg mL^–1^ in THF.

g Molar mass dispersity index obtained from SEC.

### Intramolecular Assembly of the Supramolecularly Grafted Polymers

In our next step, we followed the assembly of the polymers into intramolecularly, non-covalently crosslinked polymeric nanoparticles. In contrast to the intermolecularly crosslinked polymer network analogue, the intramolecularly crosslinked particles refer to polymers adopting a collapsed particle morphology of one or a few polymer chains. First, the protected polymers **P4**–**P9** were dissolved at 1 mg mL^–1^ in THF, which is a good solvent for the polymer backbone. THF also minimizes interparticle aggregation and allows the formation of strong hydrogen bonds after deprotection.^43^ Subsequently, the *o*-nitrobenzyl protecting group was cleaved using 365 nm light irradiation (Figure 2). The conversion of the deprotection step was monitored by ^1^H-NMR spectroscopy (Figure S10), and full deprotection was achieved after 2 h.

**Figure 2:**
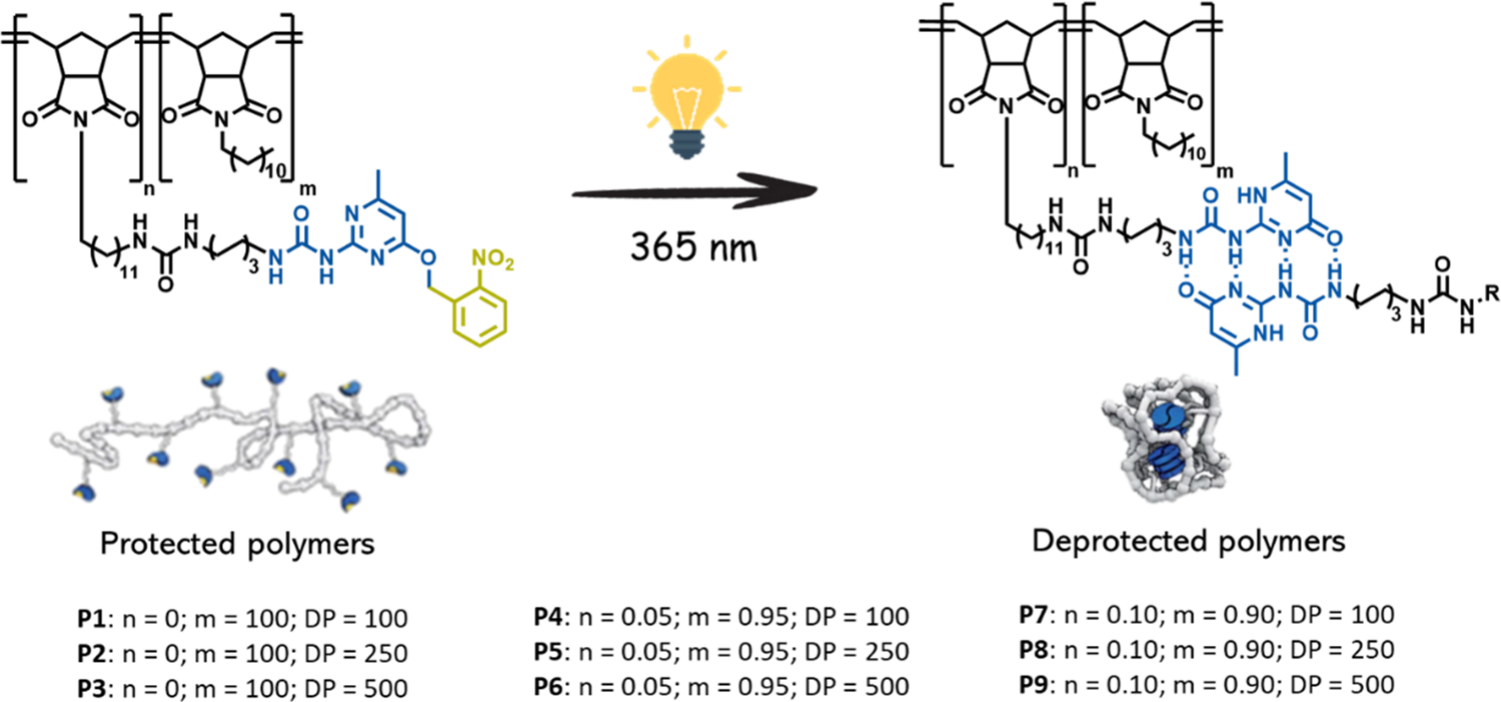
Schematic representation of the collapse of *o*-nitrobenzyl protected UPy-urea grafted polynorbornene into deprotected intramolecularly crosslinked nanoparticles.

**Figure 3:**
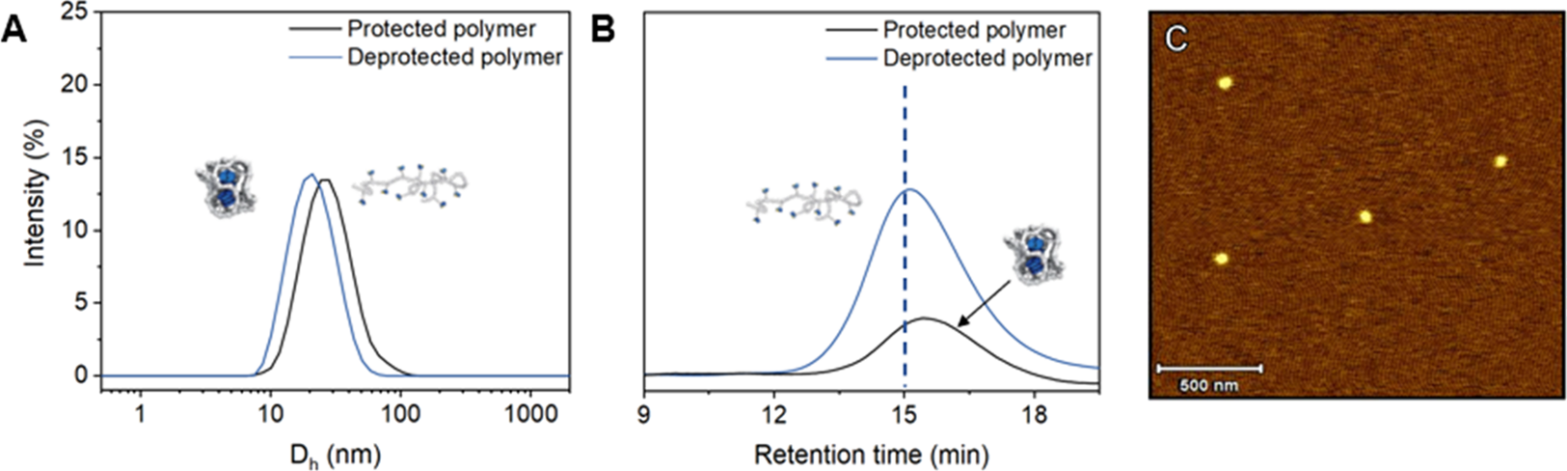
Polymer **P5** before and after deprotection by UV-light irradiation (365 nm) for 2 h at 1 mg mL^–1^ in THF demonstrating (A) a reduction in *D*_h_ (DLS) and (B) shortening of the retention time (SEC). (C) AFM height image of polymeric nanoparticles **P5** dropcasted from a 10^–8^ mg mL^–1^ solution in THF on freshly cleaved mica.

The intramolecular assembly was assessed by DLS and size exclusion chromatography (SEC) (Figure 3A,B). All deprotected polymers **P4**–**P9** exhibited a significant increase in retention time in SEC, as illustrated for polymer **P5** in Figure 3B; hence, a lower apparent molecular weight is observed, indicative of intramolecular crosslinking into (kinetically trapped) nanoparticles (Table S1). In accordance with the SEC data, DLS experiments also displayed a significant reduction of the hydrodynamic diameter *D*_h_ (13–29%), further confirming the intramolecularly crosslinked nature after UV light deprotection (polymer **P5**, Figure 3A). Furthermore, the narrow distribution suggests that the nanoparticles are uniform in size, and large multi-chain aggregates are nearly absent. To support this hypothesis, the deprotected intramolecularly assembled nanoparticle solution of **P5** was dropcasted on freshly cleaved mica and visualized by AFM. Drying effects and agglomeration of the nanoparticles were prevented by dilution of the polymer solution to 10^–8^ mg mL^–1^ in THF before dropcasting. Also in this case, uniform nanoparticles were observed (Figure 3C), which is consistent with the results from earlier works.^33,34,43^

### Processability of UPy-Urea Grafted Polymers

#### Impact of Intra- versus Intermolecular Interactions on Viscosity

To examine the contributions of intra- and intermolecular interactions on the processability of the supramolecular grafted polymers, viscosity measurements were performed at different concentrations in THF. Assuming that our polymer solutions behave as Newtonian fluids, the dynamic viscosities were measured using a falling ball viscometer followed by consecutive calculation of the specific and reduced viscosity. In the next step, the intrinsic viscosity is acquired by extrapolation of the reduced viscosity to zero (η → 0). This provides us with structural knowledge on the contribution of the deprotected polymeric nanoparticles to the overall solution viscosity and therefore the processability.

**Figure 4:**
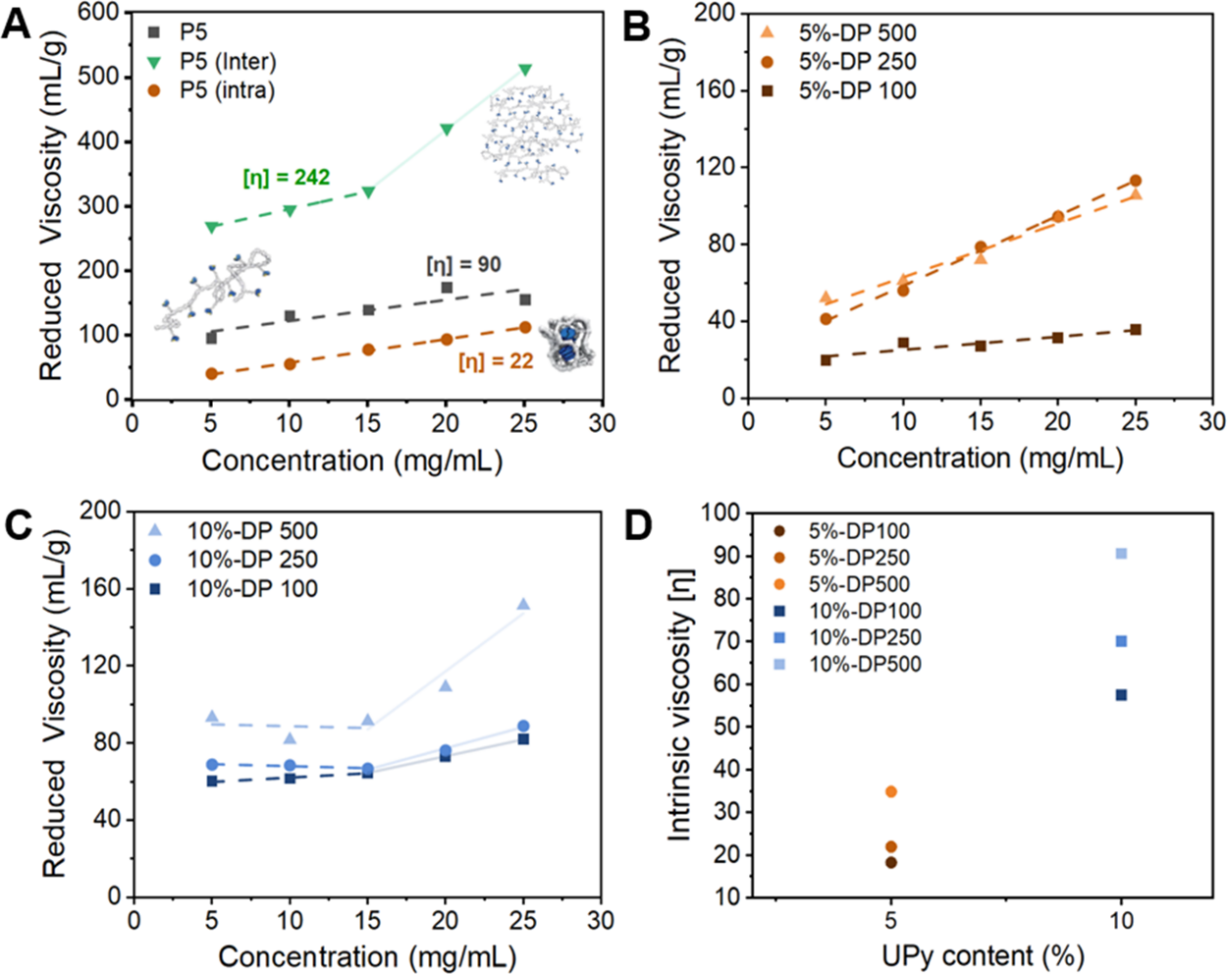
(A) Reduced viscosity plots of protected polymer **P5** (black), deprotected **P5 (inter)** (green), and deprotected **P5 (intra)** (brown). Reduced viscosity comparison of (B) deprotected **P4**–**P6 (intra)** containing 5 mol % UPy-urea and (C) deprotected **P7**–**P9 (inter)** containing 10 mol % UPy-urea. Viscosities were measured at room temperature. The corresponding extrapolations of the concentration to zero (c → 0) indicated by the dotted lines provide the intrinsic viscosities [η] of the polymer. (D) Intrinsic viscosity plotted as a function of grafting density.

First, the effects of protective group and intra- versus intermolecular crosslinking were determined by comparing the viscosities of three differently prepared polymer solutions of **P5** (Figure 4A). For clarity and clear distinction, the deprotected intramolecularly crosslinked nanoparticles and deprotected intermolecularly crosslinked network conformations will be described as **P5 (intra)** and **P5 (inter)**, respectively, from this point onward. Samples of **P5 (intra)** are prepared by deprotection of **P5** under dilute conditions (1 mg mL^–1^) promoting intramolecular collapse followed by removal of the solvent to the desired concentration for the measurements (between 5 and 25 mg mL^–1^). Samples of **P5 (inter)** were obtained by performing the deprotection at higher concentrations, 5–25 mg mL^–1^, which favors the formation of intermolecular non-covalent crosslinks. The protected polymer will be simply denoted as **P5** and is used without any further preparation steps. Figure 4A shows the reduced viscosity plotted against the concentration for **P5**, **P5 (intra)**, and **P5 (inter)**. By subsequent extrapolation (dashed lines) of the data points, the intrinsic viscosities could be extracted. As expected, the intrinsic viscosities of **P5** and **P5 (inter)** are significantly higher than those of **P5 (intra)**. Likely, the high viscosity in **P5** and **P5 (inter)** is a direct consequence of the large contribution of intermolecular interactions (such as hydrogen bonding) to viscosity. The difference in intrinsic viscosity between **P5** and **P5 (inter)** can be explained by the stronger hydrogen bonding interactions between UPy groups compared to the protected UPy units. **P5 (intra)**, in contrast, shows lower viscosities, which is rationalized by limited intermolecular interactions, a result of the intramolecular assembly of the polymer under dilute conditions. Interestingly, the intrinsic viscosity [η] (22 mL g^–1^) of the intramolecularly crosslinked **P5 (intra)** is about an order magnitude lower than that of **P5 (inter) (**[η] = 242 mL g^–1^), showcasing the potential of this approach for a better processability. Moreover, a sharp onset in viscosity was observed for concentrations higher than 15 mg mL^–1^, only occurring for **P5 (inter)**. This rapid increase in viscosity is presumably caused by exceedance of the concentration at which intermolecular interactions become more dominant for **P5 (inter)**, leading to local network formation. On the contrary, **P5** and **P5 (intra)** do not show this sharp increase at higher concentrations, most likely due to the lower association constant and high stability of intramolecular crosslinks, respectively. Consequently, the critical concentration for these polymer conformations is probably significantly higher than the concentration range measured.

#### Effect of UPy-Urea Content and Degree of Polymerization on the Nanoparticle Solution Viscosity

Besides the effect of polymer conformation and association strength, the content of UPy-urea and polymer length can also have a significant impact on the processability. Therefore, we compared the intrinsic viscosities of **P4**–**P9 (intra)** in their intramolecularly crosslinked state. For **P4**–**P6 (intra)**, containing 5 mol % of UPy-urea grafts (Figure 4B), a decrease in intrinsic viscosity is observed for shorter chain lengths, which means that smaller nanoparticles tend to be more stable and form less intermolecular interactions. In turn, longer chain lengths generally tend to result in higher viscosities. Furthermore, the number of UPy moieties per chain is higher for longer chain lengths; hence, the probability of forming intermolecular interactions will also be higher, leading to increased viscosities.

A similar trend was observed for **P7**–**P9 (intra)** (10 mol % UPy-urea), which supports our hypothesis on the effect of chain length on the viscosity (Figure 4C). Furthermore, as shown in Figure 4D, the absolute values for the intrinsic viscosity of **P7–P9 (intra)** ([η] = 50–90) are significantly higher than those for **P4**–**P6 (intra)** ([η] = 20–35). This can be attributed to the increased probability of forming intermolecular interactions during the intramolecular assembly for a higher content of UPy-urea grafts. Moreover, a sharp increase in viscosity similar to **P5 (inter)** was observed for polymers **P7**–**P9 (intra)** starting from 15 mg mL^–1^, most probably again due to crossing the concentration at which the intermolecular interactions become dominant.

Hence, the chain length and content of UPy-urea grafts play a crucial role in the ability to assemble into defined intramolecularly crosslinked nanoparticles and therefore the processability of the polymer solutions. Since intramolecular assembly seems to keep the viscosities of the solutions low, even after concentrating them, it is likely that this will significantly improve the processability of non-covalently grafted polymers.

### Intra-to-Intermolecular Assembly of Polymeric Nanoparticles

#### Visualization of Nanoparticle Intermolecular Assembly

As shown in the previous section, the use of strong and long-lived quadruple hydrogen-bonded UPy dimers limits the probability of intermolecular crosslinking between nanoparticles (kinetic trapping)^46,47^ and provide nanoparticle stability. Hence, by using these stable UPy-urea moieties, we anticipate that it is possible to bridge the gap between solution and bulk without altering the nanoparticle morphology during processing. Since molecular characterization techniques do not provide a distinction between the intra- and intermolecularly crosslinked state, we selected AFM measurements to elucidate this change in morphology going from nanoparticles to a network-like structure.

**Figure 5:**
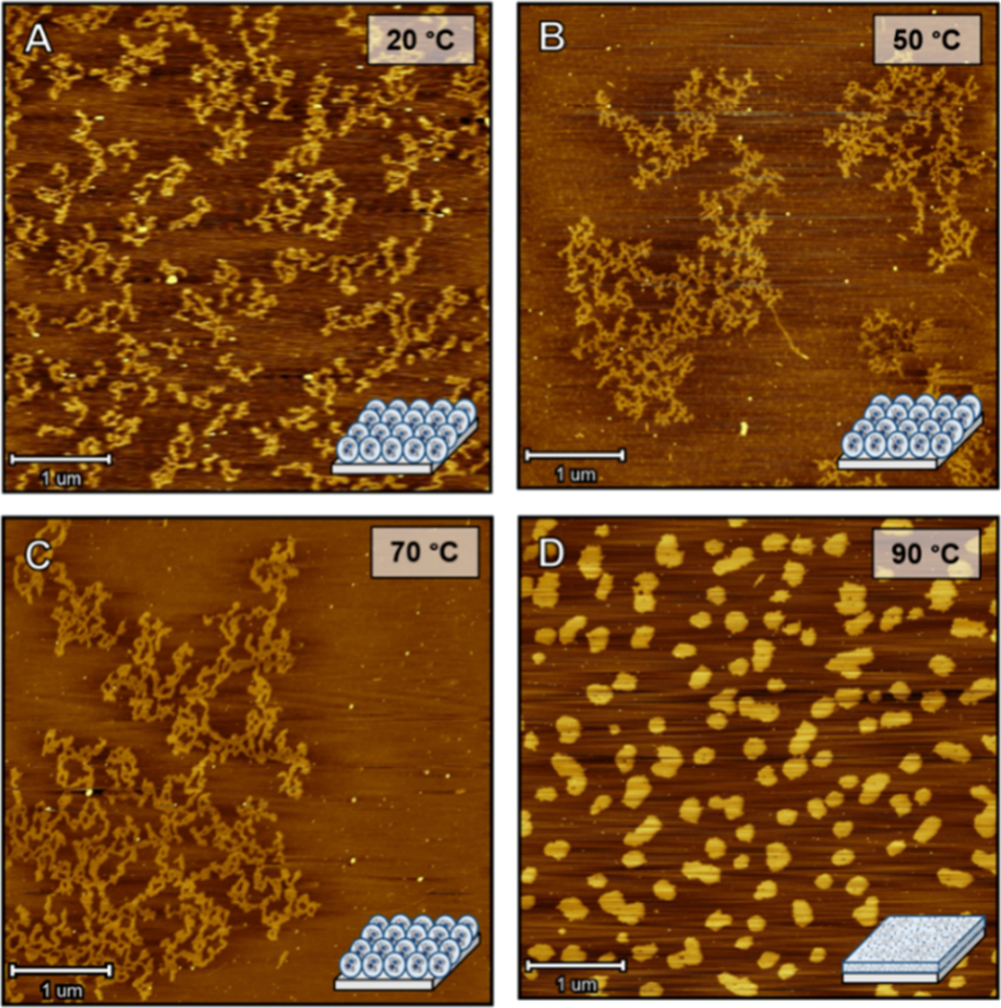
(A–D) Variable-temperature AFM images following the disassembly process of intramolecularly crosslinked polymeric nanoparticles to intermolecularly crosslinked networks occurring above the *T*_g_. The sample was dropcasted from a 10^–3^ mg/mL solution of polymer **P5 (intra)** in CHCl_3_ on freshly cleaved mica and left for 30 min at the desired temperature before taking an image.

The AFM samples were prepared by dropcasting a dilute 10^–3^ mg mL^–1^ nanoparticle solution of **P5 (intra)** on freshly cleaved mica from chloroform instead of THF. Chloroform is known to promote interparticle aggregation upon drying.^43^ As a result, we expect a morphology indicative of agglomerated nanoparticles held together by weak van der Waals interactions (Figure 5A–C), which in turn would allow us to visualize the morphology change. Next, the samples were heated to 90 °C in a stepwise fashion (10 °C/step) followed by equilibration for 30 min at each step for a temperature-induced change in morphology prior to measuring at room temperature. No significant variations in morphology were observed up till 70 °C (Figure 5C), which is still below the *T*_g_ of deprotected polymer **P5** (Table 2). Upon further heating to 85 °C and exceeding the *T*_g_ of the polymer, the morphology changed from agglomerated nanoparticles to very flat round features with larger surface areas (Figure 5D). This change can most probably be attributed to the nanoparticle disassembly because of the increased dynamics followed by the formation of an intermolecularly crosslinked network. We postulated that this process is initiated by segmental relaxation of the polymer backbone (α-relaxation) above its *T*_g_, which will be further studied in detail in the next section. It is proposed that the kinetically trapped intramolecular assembled polymers at high concentrations are transformed into the thermodynamically more stable crosslinked material. As the enthalpy of the interactions is more or less equal in both states, it is probably a delicate balance in entropy.

**Table 2:** Thermal Characterization of Polymers **P1**–**P9**

entry^a^	*T*_g,p_^c^ (°C)	*T*_g,dp_^d^ (°C)	*T*_o,1_^e^ (°C)	*T*_max,1_^e^ (°C)	*T*_o,2_^f^ (°C)	*T*_max,2_^f^ (°C)	Δ*H*_dis_^g^ (J/g)	*E*_A_^h^ (kJ/mol)
**P1**	87^b^							
**P2**	87^b^							
**P3**	85^b^							
**P4**	79	62	52	85	100	120	91	71.6 ± 2.1
**P5**	78	61	65	102	125	141	85	46.0 ± 7.4
**P6**	78	60	61	93	110	130	71	40.9 ± 1.4
**P7**	70	52	56	89	103	121	121	74.2 ± 2.2
**P8**	68	55	66	99	121	130	98	64.0 ± 5.5
**P9**	70	53	65	98	112	133	51	56.0 ± 12.5

a Polymers as depicted in Figure 2.

b Glass transition temperature of the non-functional control polymers (*T*_g,p_).

c Glass transition temperature of the *o*-nitrobenzyl protected UPy-urea grafted polymers (*T*_g,p_) in their intermolecularly crosslinked conformation.

d Glass transition temperature of the deprotected UPy-urea grafted polymers (*T*_g,dp_) in their intermolecularly crosslinked conformation.

e Onset temperatures (*T*_o,1_ and *T*_o,2_) for both exotherms observed in the first heating run of deprotected UPy-urea grafted polymers in their intramolecularly crosslinked conformation.

f Corresponding maximum temperatures (*T*_max,1_ and *T*_max,2_) for both exotherms observed in the first heating run of deprotected UPy-urea grafted polymers in their intramolecularly crosslinked conformation.

g Disassembly enthalpy of both exotherms observed in the first heating run of deprotected UPy-urea grafted polymers in their nanoparticle conformation.

h Activation energy required for disassembly of the intramolecularly crosslinked nanoparticles into an intermolecularly assembled crosslinked network.

#### Thermal Properties and Dynamics of UPy-Urea Grafted Polymers

To corroborate that disassembly of the particles is initiated by the α-relaxation process, differential scanning calorimetry (DSC) measurements were conducted on non-functionalized polymers **P1**–**P3** (Figure S11) and on both the protected polymers **P4**–**P9** and deprotected intramolecularly crosslinked polymers **P4**–**P9 (intra)**. The DSC samples for **P4**–**P9 (intra)** were prepared in a similar manner to those for the AFM measurements, wherein a 1 mg mL^–1^ nanoparticle solution in THF was concentrated to 10 mg mL^–1^. Subsequent dropcasting of aliquot amounts of solution on a glass slide and drying under reduced pressure at room temperature for at least 3 h provided us with nanoparticle films suitable for DSC measurements. A more detailed description of the sample preparation is reported in the SI.

**Figure 6:**
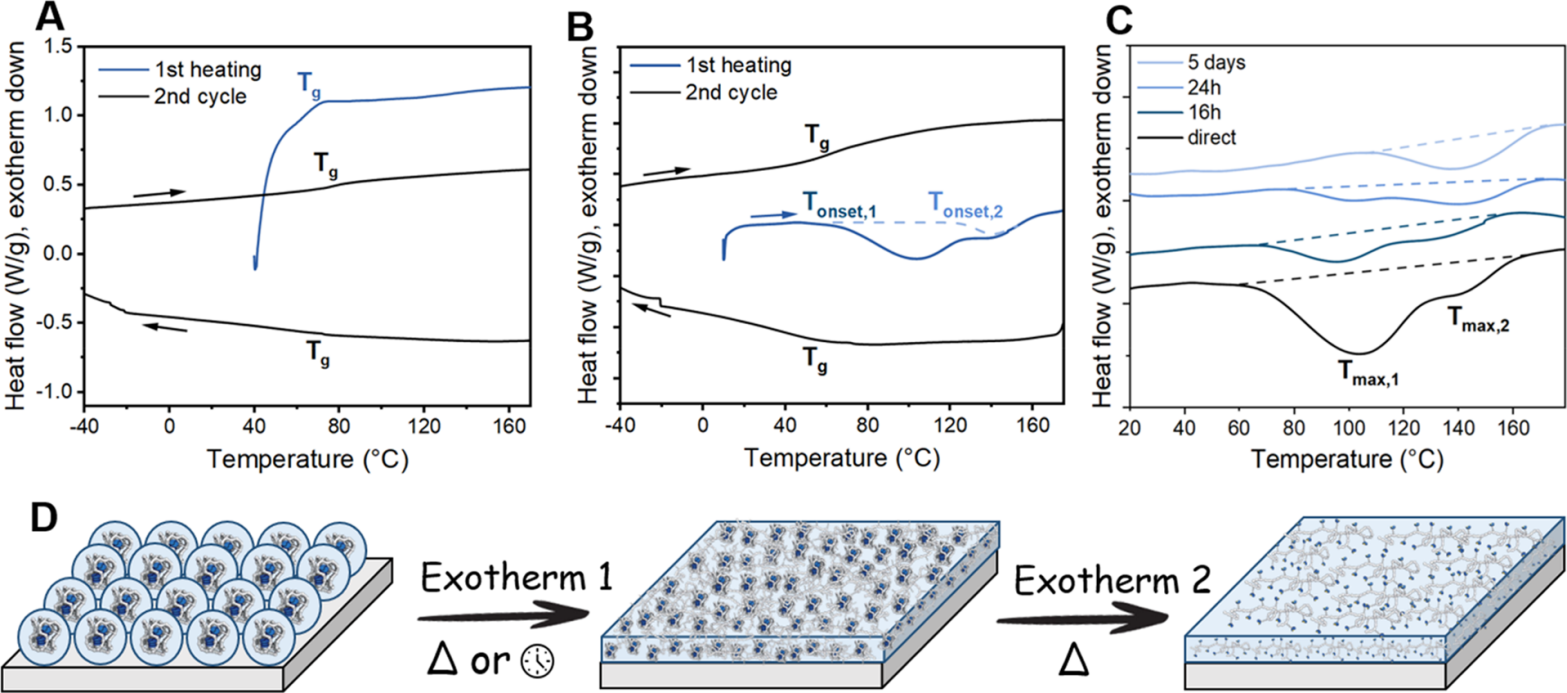
DSC traces of (A) protected polymer **P5** and (B) deprotected **P5 (intra)** (first and second heating and second cooling runs). (C) Aging effect observed for **P5 (intra)** related to α-relaxation (exotherm 1, first heating run). A temperature ramp of 10 K min^–1^ was used. (D) Illustration of the two consecutive relaxation processes leading to stepwise nanoparticle disassembly. The first transition represents α-relaxation occurring both over time (days) or with temperature (50–70 °C), while the second transition representing α*-relaxation predominantly occurs with temperatures at 100–125 °C.

In Figure 6A (and Figure S12), the first heating run and the first cycle of protected polymers **P4**–**P9** show one transition, a *T*_g_ that varies between 68 and 79 °C (Table 2) depending on the UPy-urea content. However, **P4**–**P9 (intra)** all exhibited two large overlapping exotherms, which we attribute to the disassembly of polymeric nanoparticles into an intermolecularly crosslinked network (Figure 6B). The first exotherm appears just above the *T*_g_ of the polymers and is assigned to the α-relaxation of the norbornene backbone. The second exotherm is generally observed between 120 and 150 °C, depending on the polymer length, as a shoulder of the first exotherm. This transition most probably originates from the subsequent α*-relaxation of the polymer backbone initiated by a dynamic UPy dissociation–association process, which is commonly observed in this temperature range.^48^ We assume that the nanoparticle disassembly predominantly occurs during the second exotherm since intermolecular crosslinking can only occur upon UPy dissociation.

When comparing the onset and peak temperatures at which the two relaxation processes occur for polymers **P4**–**P9 (intra)** (*T*_o,1_, *T*_max,1_, *T*_o,2_, and *T*_max,2_), no significant differences were observed (Table 2). Moreover, by comparing the *T*_g_ of non-functional polymers **P1**–**P3** and protected polymers **P4–P9**, a decreasing trend is observed upon increasing the UPy-urea content. This is presumably caused by the increased free volume created by the bulky UPy-urea grafts. A similar decreasing trend in *T*_g_ is observed for deprotected polymers **P4**–**P9 (intra)** in the second heating run (Table 2).

To confirm that the two aforementioned exotherms originate from two distinct relaxations, aging experiments were conducted on polymer **P5 (intra)**. Hereto, samples of **P5 (intra)** were prepared as described earlier and left at room temperature for 0, 16, and 24 h as well as 5 days. As observed in the thermogram in Figure 6C, the first exotherm visible at about 110 °C starts to diminish after 16 h and has completely disappeared after 5 days. On the contrary, the second exotherm is barely affected by the aging process indicative of a thermally initiated process or a process with exceptionally high relaxation times (up to months) at room temperature. This supports that the second exotherm and relaxation process are predominantly dictated by UPy dissociation, while the first exotherm is dominated by polymer backbone relaxation as shown in the cartoon in Figure 6D.

#### Nanoparticle Disassembly Kinetics

**Figure 7:**
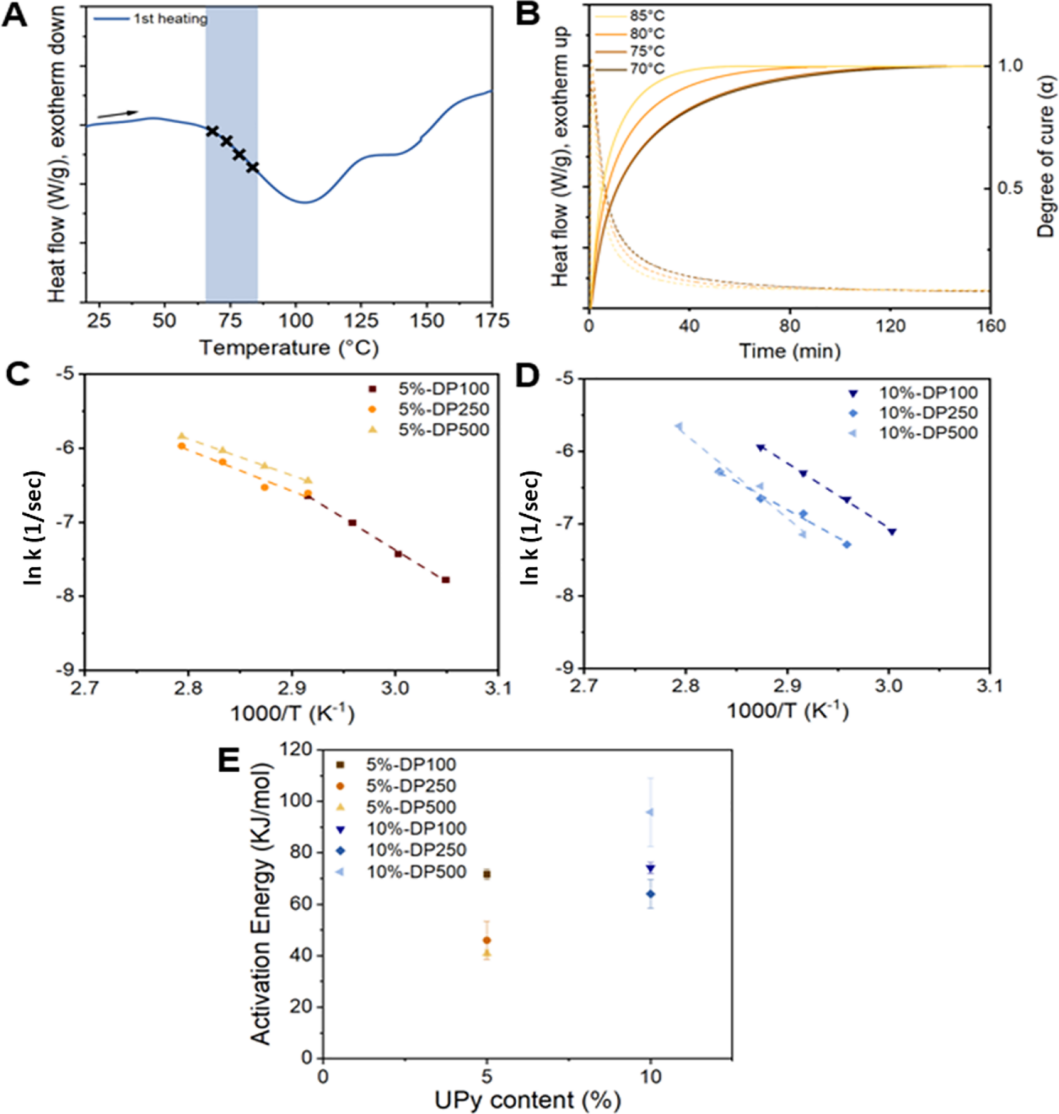
(A) Temperature determination for isothermal experiments from the first heating run of polymer **P5 (intra)** in the nanoparticle conformation. A temperature ramp of 10 K min^–1^ was used. (B) Isothermal DSC traces between 70 and 85 °C (dotted lines) with corresponding degrees of cure (α) (solid lines). Arrhenius analysis of (C) polymers **P4**–**P6 (intra)** and (D) polymers **P7**–**P9 (intra)**. (E) Activation energy for nanoparticle relaxation shown as a function of grafting density.

To get a thorough understanding of the polymer relaxation and the associated energy release, we performed kinetic DSC studies on all intramolecularly crosslinked nanoparticle films for **P4**–**P9 (intra)**. Herein, the method used is derived from the Borchardt and Daniels kinetic approach.^49^ We assumed that the nanoparticle relaxation occurs via first-order kinetics following the Arrhenius law. First, a thermogram was acquired to determine appropriate temperatures for the isothermal measurements. The selected temperatures generally range from *T*_onset_ to 50% of the height of *T*_peak_ as indicated by the black crosses in Figure 7A. Subsequently, four isotherms at the selected temperatures for each polymer were measured (dashed lines) and converted into the degree of cure as a function of time (solid lines, Figure 7B). From these conversion curves, the specific reaction constant *k*(*T*) for every temperature was determined for α = 0.1. By plotting ln[*k*(*T*)] versus 1000/*T* (Figure 7C,D) and fitting the Arrhenius law, estimated values of the activation energy for nanoparticle relaxation were obtained for polymers **P4**–**P9 (intra)** (Table 2). The results show that polymers with higher DPs result in lower activation energies, while no significant trend was observed upon increasing the content of UPy-urea grafts (5 to 10 mol %) (Figure 7E). Interestingly, the absolute activation energy values seem to be comparable to averaged values for UPy dissociation^50^ and viscous flow of conventional polymers,^51^ arguably indicating the presence of a synergistic process. However, in-depth mechanistic studies would be required to support this hypothesis.

#### Redissolution of Intra- and Intermolecularly Crosslinked Polymer Films

**Figure 8:**
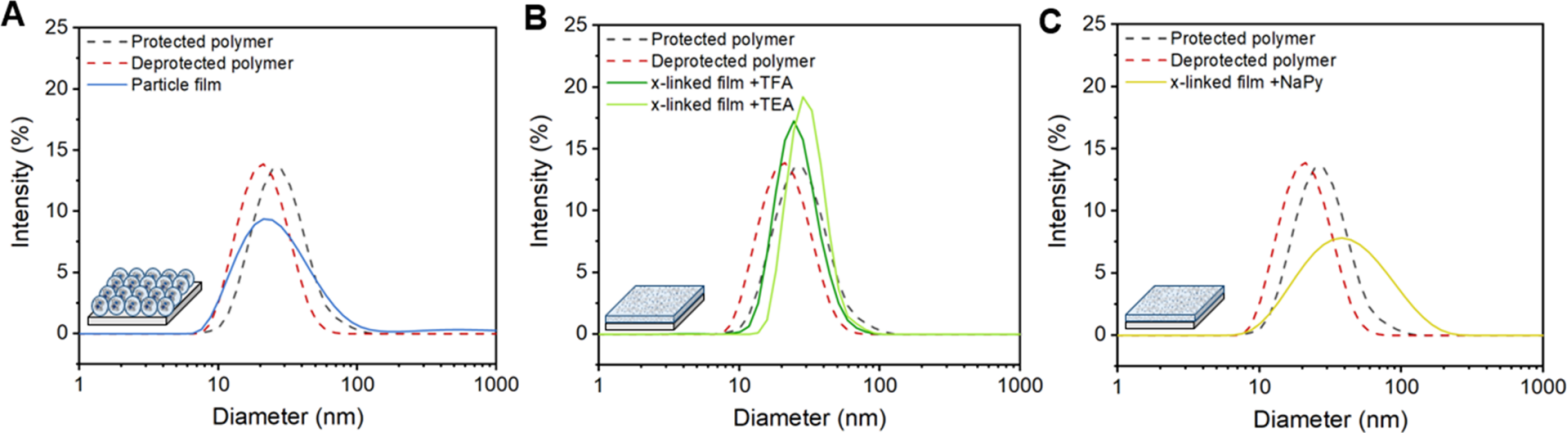
DLS intensity distributions as a function of the hydrodynamic diameter (*D*_h_) of redissolved intra- and intermolecularly crosslinked films of polymer **P5** upon 2 min of sonication at 1 mg mL^–1^ in THF. (A) Upon redissolution of the nanoparticle film **P5 (intra)**; (B) after redissolution of an intermolecular crosslinked film **P5 (inter)** sequentially followed by addition of 100 μL of TFA and by 100 μL of TEA; and (C) after redissolution of an intermolecular crosslinked film **P5 (inter)** upon addition of an excess of naphthyridine (NaPy).

The dynamic nature of the supramolecular networks enables recycling of the polymers, either thermally or by redissolution. This prompted us to test the reversibility of this process by reobtaining uniform nanoparticle solutions from both the intramolecularly crosslinked nanoparticle and the intermolecularly crosslinked network films. Herein, films of **P5 (intra)** as prepared for DSC analysis were sonicated for 2 min in an excess of THF. The quantity of solvent was then reduced to reach a concentration of 1 mg mL^–1^ after which they were subjected to DLS analysis and compared with the initial intramolecular assembly measurements (Figure 8).

After redissolution of the nanoparticle film of **P5 (intra)** in THF (Figure S14) and sonication for 2 min, the nanoparticles fully recover to their initial sizes and show a uniform yet slightly broader size distribution, possibly due to nanoparticle aggregation (Figure 8A, blue). Contrarily, the crosslinked polymer film of **P5 (inter)** could not be redissolved due to the highly crosslinked nature of the material (Figure S15A). However, upon addition of a small amount of trifluoroacetic acid (TFA), which disrupts the hydrogen bonding of the UPy dimers,^34^ the crosslinked film redissolved (Figure S15B). Subsequent DLS analysis revealed similarly sized polymeric nanoparticles with a narrow size distribution presumably in their random coil conformation due to UPy dimer inhibition by TFA (Figure 8B, dark green). Unfortunately, consecutive neutralization of TFA upon addition of an aliquot amount of triethylamine (TEA) as a base did not result in smaller nanoparticles but slightly larger-sized nanoparticles (Figure 8B, light green). An explanation could be that interparticle interactions are promoted by the presence of the TEA-TFA salt. Nevertheless, redissolution of the crosslinked polymer film **P5 (inter)** and thus reversibility of the system were possible. Furthermore, we tried to redissolve the intermolecularly crosslinked film of **P5 (inter)** by adding an excess of naphthyridine (NaPy), which forms a heterocomplex with UPy and would thus act as a dimerization inhibitor.^52^ However, only partial redissolution could be acquired (Figure S15C). Possibly, the equilibrium that is formed between the homo- and heterodimers could have resulted in a complex mixture, eventually leading to a broader size distribution (Figure 8C, yellow). Hence, the use of NaPy is a less suitable approach to achieve reversibility in this system.

## Conclusions

In this work, we successfully presented a novel approach showing the enhanced processability and stability of supramolecular grafted polymers by controlling the intra- and intermolecular interactions. Hereby, we used the concentration as a tool for intramolecular polymer chain collapse, resulting in significantly lower viscosities than their intermolecularly crosslinked analogues. In general, increasing the chain length of the polymers and content of UPy-urea increased the propensity for intermolecular interactions and thus lead to higher viscosities. After processing the nanoparticles into the bulk, AFM analysis provided a first indication of nanoparticle disassembly indicated by a change in morphology upon exceedance of the *T*_g_ of the grafted polymers. Subsequent DSC analysis demonstrated the formation of stable nanoparticle films and revealed two exothermic relaxation processes during the first heating run in contrast to the processed random coil polymers. Herein, the two processes are related to the α- and α*-relaxation, in which the latter provides the intramolecularly trapped nanoparticles with sufficient energy to form an intermolecular crosslinked network. Further in-depth DSC analysis on the kinetics of nanoparticle relaxation in the bulk revealed a similar trend to that in solution, demonstrating the effect of chain length and UPy content on the activation barrier. Ultimately, by redissolution of intermolecularly crosslinked polymer films in the presence of small amounts of additive, we could show reversibility in this system. These fundamental and structural insights into the interplay of inter- and intramolecular crosslinking broaden the scope of applications for supramolecular polymers and increase their potential in the field of sustainability. We envision that this novel processing approach of non-covalently grafted polymers will lead to further development in the field of reprocessable plastics.

## Supporting Information Available

Experimental details, materials and methods, detailed synthetic procedures of all monomers, polymers, and intermediates, NMR spectra of final monomers and polymers, FTIR spectra of all polymers, NMR spectra of intramolecular folding of polymer **P5**, variable temperature and isothermal DSC traces of polymers, and photographs of redissolution studies (PDF)
