# Supplementary material for: Controlling the Processability and Stability of Supramolecular Polymers Using the Interplay of Intra- and Intermolecular Interactions
Source: Macromolecules. 2022 Jul 27;55(15):6820–9. doi: 10.1021/acs.macromol.2c00976 (PMC9367003; doi:10.1021/acs.macromol.2c00976)
Supplement: Supplementary file 1 — ma2c00976_si_001.pdf [file ma2c00976_si_001.pdf]

Supplementary information

Controlling the processability and stability of supramolecular polymers using the interplay of intra- and intermolecular interactions

Joost J. B. v. d. Tol, Ghislaine Vantomme, Anja R. A. Palmans, E. W. Meijer.*

Institute for Complex Molecular Systems and Laboratory of Macromolecular and Organic Chemistry, Eindhoven University of Technology, P.O. Box 513, 5600 MB Eindhoven, The Netherlands.

* Corresponding author e-mail: e.w.meijer@tue.nl

Table of Contents

1. Materials and methods 2
2. Sample preparation 4
3. Synthetic procedures 5
4. NMR Spectra of final monomers and polymers. 10
5. FTIR spectra polymers P1–P9 14
6. NMR spectra of intramolecular folding of P5 15
7. Thermal properties of polymer P1–P9 17
8. Redissolution of intra- and intermolecularly crosslinked polymer films of P5 19

1. Materials and methods

All reagents were purchased from commercial resources and used without further purification. Monodisperse di- and mono-hydride dimethylsiloxane oligomers were obtained according to literature procedure.[1,2] Solvents were purchased from Biosolve and dry solvents were obtained using the MBraun solvent purification system (MB SPS-800). Oven-dried glassware (120 °C) was used for all reactions carried out under argon atmosphere. Deuterated compounds were obtained from Cambridge Isotopes Laboratories. Reactions were followed by thin-layer chromatography (TLC) using Merck's 60-F_{254} silica gel plates and when necessary visualized by potassium permanganate ($KMnO_4$) stain.

NMR spectra were recorded using a Varian Mercury Vx 400 MHz (^{1}H-NMR using 400 MHz and ^{13}C-NMR using 100 MHz). Proton and carbon chemical shifts are reported in ppm (δ) downfield from trimethylsilane (TMS) using the deuterated solvent resonance frequency as internal standard. Peak multiplicities are abbreviated as s: singlet; d: doublet; t: triplet; q: quartet; p: pentet; m: multiplet; dd: double doublet; dt: double triplet and dq: double quartet.

Matrix assisted laser absorption/ionization mass time of flight (MALDI-TOF) measurements were performed on a Bruker Autoflex Speed using α-cyano-4-hydroxycinnamic acid (CHCA) and *trans*-2-[3-(4-*tert*-butylphenyl)-2-methyl-2-propenylidene]malononitrile (DCBT) as matrices.

THF-SEC measurements were carried out in PL-GPC-50 plus from Polymer Laboratories (Agilent Technologies) with the refractive index detector working in THF containing 10 mM LiBr at 50 °C at a constant flow rate of 1mL min^{-1} on a Shodex GPC-KD-804 column (exclusion limit = 400 000 Da; 0.8 cmi.d. × 300 mL), which was calibrated with polystyrene (PS) samples with a range from 282-77350 Da (Polymer Laboratories-Agilent Technologies). Samples were prepared at 1 mg mL^{-1} in THF and filtered through 0.2 μm Whatman Anatop 10 filters prior to injection.

Differential scanning calorimetry (DSC) data were collected on a DSC Q2000 from TA instruments, calibrated with an indium standard. The samples (2–8 mg) were weighed directly into aluminum pans and hermetically sealed. Freshly prepared polymer samples were initially heated to 180 °C and then subjected to two cooling/heating cycles from −50 °C to 180 °C with a rate of 10 K min^{-1}. The data that is presented, represents the first and second heating and second cooling cycle. Polymeric particle samples for aging studies were first subjected to a predetermined incubation time prior to the measurement. Isothermals were collected by loading the freshly prepared polymeric particle samples at the desired temperature after which the measurement is started directly.

Atomic Force Microscopy (AFM) images were taken using an Asylum Research MFP-3D Origin mounted on an anti-vibration stage surrounded by an acoustic chamber. Silicon probes (NCSTR-50) with a tip height of 10–15 µm and radius of <10 nm were used to take images in attractive tapping-mode with a 1024x1024 resolution and 1.09 Hz scan rate. Samples were prepared by dropcasting 40 µL from a desired concentration of deprotected particle solution in either THF or $CHCl_3$ onto freshly cleaved 1x1 cm^2 sized Mica followed by overnight drying in air. Images were extracted using the software Gwyddion v2.50.

Dynamic light scattering (DLS) measurements were conducted using a Malvern µV zetasizer equipped with an 830 nm laser and a scattering angle of 90°. A fluorescence cell with a 1 mm path length was used for the measurements. The hydrodynamic radius determined from these measurements is an apparent radius, hence R_h is equal to $R_{h,\,90°}{}^{app}$. The temperature-dependent viscosity of tetrahydrofuran was obtained from the machine catalogues.

Viscosity measurements were performed on a Lovis 2000 M/ME rolling ball viscometer equipped with a 5.5 mm capillary using a gold coated ball to prevent interactions with the solute. Prior to the measurements, the viscometer is calibrated according to ISO 17025 with a standard (APS6). All dynamic viscosity measurements were performed at 20 °C by measuring the long distance time at an angle of 45°. The reference dynamic viscosity of THF was acquired from literature. Subsequent reduced viscosities were obtained by sequentially calculating the relative and specific viscosity from the dynamic viscosity followed by dividing through concentration.

2. Sample preparation

DLS measurements

Polymer samples **P1–P9** were prepared by dissolving 1 mg of polymer in 1 mL of THF followed by filtration through a 200 nm PTFE filter. Samples **P1–P9 (intra)** were prepared by dissolving 1 mg of polymer in 1 mL of THF followed by a deprotection step using 365 nm light while vigorously stirring for at least 2 h. Subsequently, the particle solution was filtered through a 200 nm PTFE filter prior to the measurements.

Viscosity measurements

Sample **P5** was prepared by dissolving the required amount of polymer in 1 mL THF to reach the desired concentration.

Sample **P5 (inter)** was prepared by dissolving the required amount of polymer in 1 mL THF to reach the desired concentration for the measurement. Subsequently, these polymer solutions were subjected to 365 nm light while vigorously stirring for at least 2 h prior to the measurement.

Samples **P4–P9 (intra)** were prepared by dissolving the required amount of polymer at a concentration of 1 mg mL^{-1} followed by a deprotection step using 365 nm light while vigorously stirring for 2 h. After deprotection, the polymer solutions were brought to the desired concentration for viscosity measurements by applying a N_2 flow on top of the solution to evaporate the solvent from the same vial. A gentle flow is used in order to prevent premature temperature-induced aggregation.

DSC measurements

DSC samples of polymers **P1–P9** were prepared as usual, hence the material is used as it is. DSC samples of the intramolecularly crosslinked polymeric nanoparticles **P1–P9 (intra)** were prepared by dissolving 10 mg of polymer in a 15 mL vial with 10 mL THF followed by a deprotection step using 365 nm light while vigorously stirring for 2 h. After folding, the particle solutions are concentrated to 10 mg mL^{-1} (1 mL solvent left) using a N_2 flow on top of the solution to evaporate the solvent. A gentle flow is used in order to prevent premature temperature-induced aggregation. Subsequently, twice 0.5 mL of particle solution is dropcasted onto two different glass microscope slides covering the entire surface followed by 15 min drying in air and for 3 h vacuum drying at room temperature prior to the DSC measurement. Finally, the dried powder-like coatings (Figure **S13**) are scraped off the glass slide using a sharp razor blade and used for DSC measurements.

3. Synthetic procedures

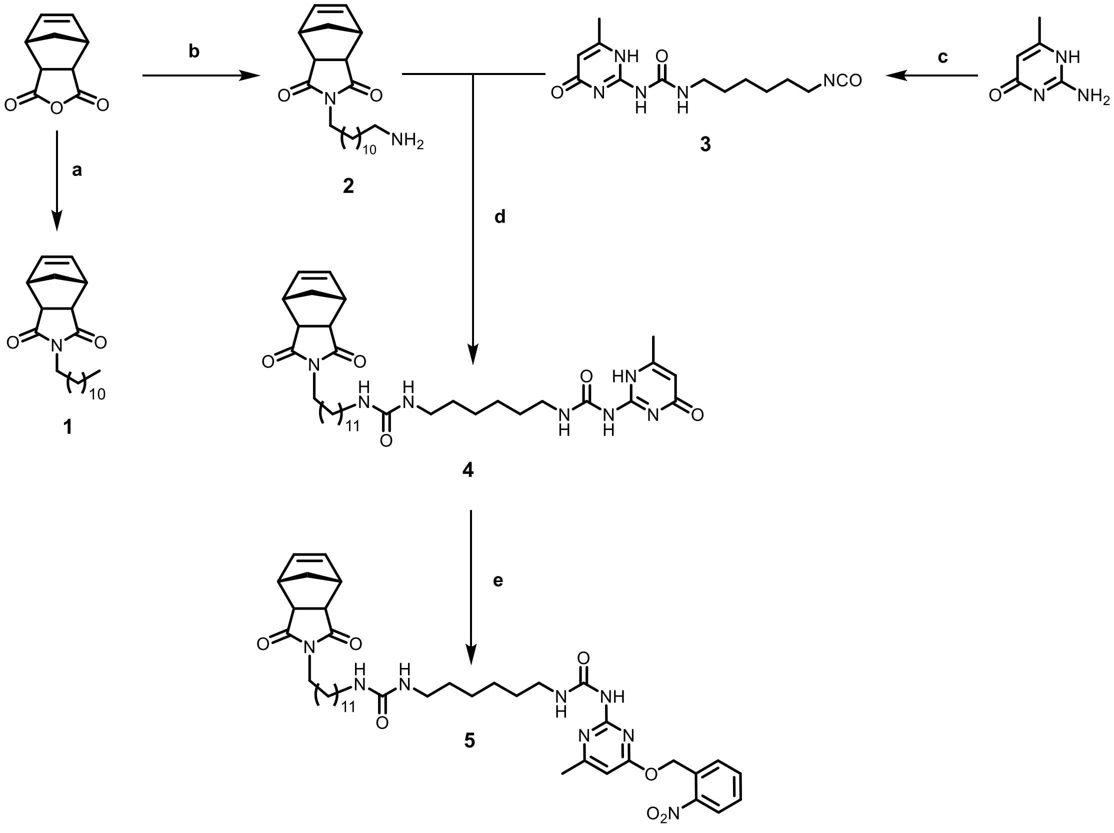

Scheme S1. Synthesis of norbornene monomers **1** and **5**. Reaction conditions: a) Toluene, dodecylamine, TEA, 100 °C, 16 h (58%). b) Toluene, diaminododecane, TEA, 100 °C, 16 h (34%). c) Hexamethylene diisocyanate, 100 °C, 16 h (91.4%). d) $CHCl_3$, DBTL, 60 °C i) 16 h ii) 60 Å silica gel, 3 h. e) DMF, K_2CO_3, 2-nitrobenzylchloride, 80 °C, 24 h (35% over steps d and e).

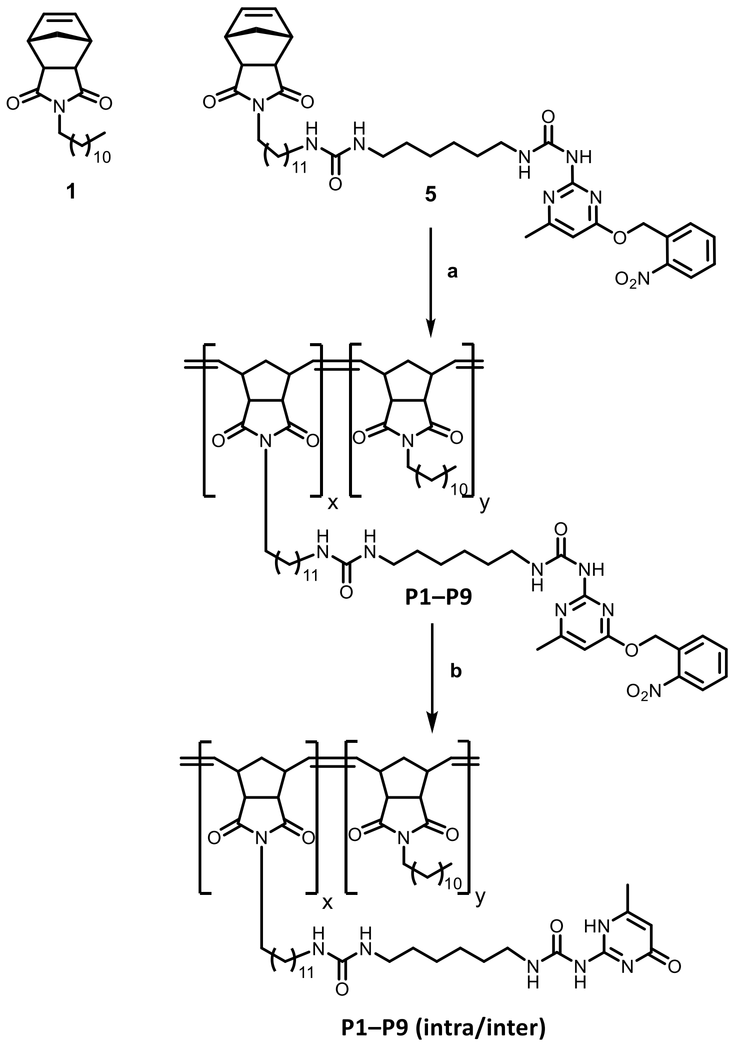

Scheme S2. Synthesis of norbornene polymers **P1–P9** and **P1–P9 (intra/inter)**. Reaction conditions: a) DCM, 2nd generation Grubbs catalyst, RT, 16 h (dichloroethane, 50 °C for **P6** and **P9**). b) THF, 365 nm light, 1 mg mL^{-1} RT, 16 h.

(4R,7S)-2-dodecyl-3a,4,7,7a-tetrahydro-1H-4,7-methanoisoindole-1,3(2H)-dione (1)

Cis-5-Norbornene-endo-2,3-dicarboxylic anhydride (10.0 g, 60.9 mmol), dodecylamine (3.96 g, 67.0 mmol, 1.1 eq) and triethylamine (7.09 g, 70.1 mmol, 1.15 eq) in 200 mL of toluene were added to a 500 mL round bottom flask. The resulting suspension was purged with argon and refluxed using a Dean-Stark apparatus while vigorously stirring. After 16 h, the clear yellowish solution was allowed to cool down followed by solvent removal under reduced pressure. The crude product was then redissolved in 200 mL DCM and washed sequentially with a 100 mL 0.2 M HCl solution, 100 mL saturated $NaHCO_3$ and 100 mL brine. The organic phase was collected and dried over $MgSO_4$ followed by solvent removal under reduced pressure. The crude product was dissolved in DCM and purified by automated column chromatography using Heptane/EtOAc (gradient 100/0 to 60/40) as eluent yielding pure **1** as a transparent viscous liquid (11.7 g, 58 %). ^{1}H NMR (400 MHz, Chloroform-*d*) δ = 6.09 (t, 2H), 3.38 (m, 2H), 3.31 (t, ^3J = 7.5 Hz, 2H), 3.24 (m, ^3J = 2.6 Hz, 2H), 1.73 (d, ^3J = 8.8 Hz, 1H), 1.54 (d, ^3J = 8.7 Hz, 1H), 1.42 (p, ^3J = 7.3 Hz, 2H), 1.24 (m, 18H), 0.88 (t, ^3J = 6.6 Hz, 3H). ^{13}C NMR (100 MHz, Chloroform-*d*) δ = 177.69, 134.38, 52.17, 45.68, 44.87, 38.43, 31.88, 29.56, 29.30, 29.12, 27.79, 26.86, 22.65, 14.09 ppm. MS (MALDI-TOF): *m/z* calc for $C_{21}H_{33}NO_2^+$: 331.25 $[M+H]^+$; found 332.27.

(4R,7S)-2-(12-aminododecyl)-3a,4,7,7a-tetrahydro-1H-4,7-methanoisoindole-1,3(2H)-dione (2)

Cis-5-Norbornene-endo-2,3-dicarboxylic anhydride (2.5 g, 15.2 mmol), 1,12-diaminododecane (12.21 g, 60.9 mmol, 4 eq) and triethylamine (6.32 g, 62.4 mmol, 4.1 eq) in 50 mL of toluene were added to a 100 mL round bottom flask. The resulting suspension was purged with argon and refluxed using a Dean-Stark apparatus while vigorously stirring. After 16 h, the clear yellowish solution was allowed to cool down followed by solvent removal under reduced pressure. The crude product was then redissolved in 100 mL DCM and washed sequentially with 0.2 M HCl solution (4x 50 mL), 100 mL saturated $NaHCO_3$ and 100 mL brine. The organic phase was collected and dried over $MgSO_4$ followed by solvent removal under reduced pressure resulting. The crude product was dissolved in $CHCl_3$ and purified by automated column chromatography using $CHCl_3$/MeOH (gradient 100/0 to 90/10) as eluent yielding pure **2** as a white solid (1.78 g, 34 %). M.p. 98 °C. ^{1}H NMR (400 MHz, Chloroform-*d*) δ = 6.09 (t, ^3J = 1.9 Hz, 2H), 3.38 (m, 2H), 3.31 (t, ^3J = 7.4 Hz, 2H), 3.24 (dd, ^3J = 3.0, 1.5 Hz, 2H), 2.97 (m, 2H), 1.76 (p, ^3J = 7.6 Hz, 2H), 1.73 (dt, ^3J = 8.6, 1.5 Hz, 1H), 1.54 (dt, ^3J = 8.7, 1.6 Hz, 1H), 1.41 (m, 2H), 1.27 (m, 2H), 1.24 (m, 16H). ^{13}C NMR (100 MHz, Chloroform-*d*) δ = 177.78, 134.41, 52.22, 45.72, 44.90, 39.98, 38.46, 29.46, 29.35, 29.13, 28.96, 27.80, 27.75, 26.88, 26.49 ppm. MS (MALDI-TOF): *m/z* calc for $C_{21}H_{34}N_2O_2^+$: 346.21 $[M+H]^+$; found 347.24.

1-(6-isocyanatohexyl)-3-(6-methyl-4-oxo-1,4-dihydropyrimidin-2-yl)urea (3)

2-amino-6-methylpyrimidin-4(1H)-one (2 g, 15.98 mmol) and hexamethylene diisocyanate (16.13 g, 95.9 mmol, 6 eq) were added to a 100 mL round bottom flask. The resulting suspension was purged with argon for 15 min and heated to 100 °C for 16 h. The crude mixture was allowed to cool down after which 50 mL of pentane was added. The resulting precipitate was vacuum filtered, washed with pentane and vacuum dried for 16 h at 60 °C yielding the pure **3** as white a white solid. (4.29 g, 91.4 %.). M.p. 215 °C. ^{1}H NMR (400 MHz, Chloroform-*d*) δ = 13.11 (s, 1H), 11.86 (s, 1H), 10.18 (s, 1H), 5.82 (s, 1H), 3.28 (t, ^3J = 6.7 Hz, 2H), 3.26 (q, ^3J = 6.5 Hz, 2H), 2.23 (s, 3H), 1.67 – 1.54 (m, 4H), 1.47 – 1.33 (m, 4H). ^{13}C NMR (100 MHz, Chloroform-*d*) δ = 187.15, 173.09, 156.61, 154.72, 148.29, 106.71, 42.89, 39.79, 31.20, 29.31, 26.24, 26.18, 18.95. MS (MALDI-TOF): *m/z* calc for $C_{13}H_{19}N_5O_3^+$: 293.15 $[M+H]^+$; found 294.11.

1-(12-((4R,7S)-1,3-dioxo-1,3,3a,4,7,7a-hexahydro-2H-4,7-methanoisoindol-2-yl)dodecyl)-3-(6-(3-(6-methyl-4-oxo-1,4-dihydropyrimidin-2-yl)ureido)hexyl)urea (4)

Compound **2** (1.5 g, 6.81 mmol) and compound **3** (2.4 g, 8.2 mmol, 1.2 eq) in 50 mL $CHCl_3$ were added to a 100 mL round bottom flask, purged with argon and heated to 50 °C for 16 h. The resulting suspension was then allowed to cool down and the solvent was removed under reduced pressure. Due to poor solubility of **4** in any solvent, the crude mixture was used without any further purification in the next step.

1-(12-((4R,7S)-1,3-dioxo-1,3,3a,4,7,7a-hexahydro-2H-4,7-methanoisoindol-2-yl)dodecyl)-3-(6-(3-(4-methyl-6-((2-nitrobenzyl)oxy)pyrimidin-2-yl)ureido)hexyl)urea (5)

To a suspension of crude mixture of **4** (2.5 g, 3.91 mmol) in 100 mL of DMF, K_2CO_3 (1.8 g, 7.0 mmol, 1.8 eq) was added and the solution was purged with argon. Subsequently, 2-nitrobenzylchloride (938.6 mg, 5.47 mmol, 1.4 eq) was added to the suspension and heated to 80 °C for 16 h. After the reaction, solvent was evaporated under reduced pressure, redissolved in 50 mL $CHCl_3$ and washed with water (3x 25 mL) and brine. The organic phase was collected, dried over Na_2SO_4 and vacuum filtered to yield a clear yellow solution. The solvent was removed under reduced pressure, redissolved in DCM and purified by automated column chromatography using DCM/MeOH (gradient 100/0 to 80/20) as eluent yielding pure **5** as a pale yellow crystalline solid (1.06 g, 35 % over two steps). No melting point observed. ^{1}H-NMR (400 MHz, Chloroform-*d*): δ = 9.13 (s, 1H), 8.12 (d, ^3J = 8.2 Hz, 1H), 7.67 – 7.62 (m, 2H), 7.50 (m, 1H), 7.11 (s, 1H), 6.28 (s, 1H), 6.09 (t, ^3J = 1.9 Hz, 2H), 5.73 (s, 2H), 4.74 (t, ^3J = 5.7 Hz, 1H), 4.50 (t, ^3J = 5.7 Hz, 1H), 3.45 – 3.03 (m, 12H), 2.37 (s, 3H), 1.71 (m, 2H), 1.60 – 1.20 (m, 28H). ^{13}C NMR (100 MHz, Chloroform-*d*) δ = 177.80, 169.56, 158.44, 157.27, 154.29, 147.58, 134.41, 133.79, 132.39, 128.95, 128.77, 125.04, 100.29, 64.72, 52.22, 45.72, 44.90, 40.59, 40.10, 39.49, 38.475830.29, 29.88, 29.74, 29.52, 29.48, 29.43, 29.34, 29.11, 27.80, 26.92, 26.87, 26.23, 26.10, 23.81 ppm. MS (MALDI-TOF): *m/z* calcd for $C_{41}H_{58}N_8O_7^+$: 774.44 $[M+Na]^+$; found 797.46.

General procedure for the synthesis of polymers P1-3

Compound **1** (125.9 mg, 0.38 mmol) in 1 mL dry DCM was transferred into a 5 mL Schlenk tube followed by degassing the clear solution for at least 15 minutes using argon. A separate solution of 2nd generation Grubbs catalyst (3.23 mg, 3.8 µmol, 0.01 eq) in dry degassed 0.1 mL DCM was then added to the vigorously stirring polymer solution. After 8 hours the polymerization was quenched with an excess vinyl ethyl ether (1 mL) and was allowed to stir for an additional 30 minutes. The reaction mixture was then concentrated by removal of excess solvent and precipitated into 200 mL of diethyl ether. The precipitate was vacuum filtered followed by drying at 50 °C under reduced pressure for 4 h yielding pure **P1** (103.5 mg, 82 %) as a fibrous paper like white solid. ^{1}H-NMR (400 MHz, Chloroform-*d*): δ = 5.78 – 5.50 (m, 2H), 3.47 (m, 2H), 3.31 – 2.82 (m, 4H), 2.01 – 1.22 (m, 22H), 0.87 (t, ^3J = 6.7 Hz, 3H). SEC: M_n = 51 kDa, PDI = 2.15.

Starting from **compound 1** (125.3 mg, 0.38 mmol) and 2nd generation Grubbs catalyst 1.29 mg, 1.52 µmol, 0.004 eq), pure **P2** (64 mg, 67 %) was obtained as a fibrous paper like white solid using the general procedure as described for **P1**. The NMR data was identical to that of **P1**. SEC: M_n = 76 kDa, PDI = 2.16.

Starting from **compound 1** (124.9, 0.37 mmol) and 2nd generation Grubbs catalyst (0.645 mg, 0.76 µmol, 0.002 eq), pure **P3** (99.5 mg, 79 %) was obtained as a fibrous paper like white solid using the general procedure as described for **P1**. The NMR data was identical to that of **P1**. SEC: M_n = 135 kDa, PDI = 2.06.

General procedure for the synthesis of polymers P4-9

Compound **1** (125.0 mg, 0.38 mmol) and compound **5** (15.5 mg, 0.02 mmol, 0.05 eq) were dissolved in 1.5 mL dry DCM and transferred into a 5 mL Schlenk tube followed by degassing the clear solution for at least 15 minutes using argon. A separate solution of 2nd generation Grubbs catalyst (3.19 mg, 0.01 eq, 3.8 µmol) in dry degassed 0.1 mL DCM was then added to the vigorously stirring polymer solution. After 16 hours the polymerization was quenched with an excess vinyl ethyl ether (1 mL) and was allowed to stir for an additional 30 minutes. The reaction mixture was then concentrated by removal of excess solvent and precipitated into 200 mL of hexanes. The precipitate was vacuum filtered followed by drying at 50 °C under reduced pressure for 4 h yielding pure **P4** (104 mg, 73 %) as a fibrous paper like white solid. ^{1}H-NMR (400 MHz, Chloroform-*d*): δ = 9.13 (s, 1H), 8.12 (d, ^{3}J = 8.2 Hz, 1H), 7.65 (m, 2H), 7.51 (m, 1H), 7.02 – 6.98 (s, 1H), 6.28 (s, 1H), 5.79 – 5.46 (broad m, 40H), 4.70 – 4.35 (broad m, 2H), 3.54 – 2.84 (broad m, 132H), 2.37 (s, 3H), 2.07 - 1.12 (broad m, 448H), 0.87 (t, ^{3}J = 6.7 Hz, 60H). SEC: M_n = 55 kDa, PDI = 1.81.

P5: Starting from compound **1** (996 mg, 3.01 mmol), compound **5** (122.6 mg, 0.16 mmol, 0.05 eq) and 2nd generation Grubbs catalyst (10.2 mg, 12 µmol, 0.004 eq), pure **P5** (1019 mg, 91 %) was obtained as a fibrous paper like white solid using the general procedure as described for **P4**. The NMR data was identical to that of **P4**. SEC: M_n = 84 kDa, PDI = 1.7.

P6: Starting from compound **1** (126.0 mg, 0.38 mmol), compound **5** (15.2 mg, 0.19 mmol, 0.05 eq) and 2nd generation Grubbs catalyst (0.638 mg, 0.74 µmol, 0.002 eq), pure **P6** (93 mg, 66 %) was obtained as a fibrous paper like white solid using the general procedure as described for **P4**. However, in this particular case 1.5 mL of dry, degassed DCE was used and the reaction was allowed to vigorously stir for 16 h at 50 °C. The NMR data was identical to that of **P4**. SEC: M_n = 187 kDa, PDI = 1.7.

P7: Starting from compound **1** (126.5 mg, 0.39 mmol), compound **5** (32.7 mg, 0.42 mmol, 0.1 eq) and 2nd generation Grubbs catalyst 3.19 mg, 3.75 µmol, 0.01 eq), pure **P7** (102 mg, 65 %) was obtained as a fibrous paper like white solid using the general procedure as described for **P4**. ^{1}H-NMR (400 MHz, Chloroform-*d*): δ = 9.13 (s, 1H), 8.12 (d, ^{3}J = 8.1 Hz, 1H), 7.65 (m, 2H), 7.50 (m, 1H), 7.02-6.98 (s, 1H), 6.28 (s, 1H), 5.79 – 5.41 (broad m, 40H), 4.97 - 4.28 (broad m, 2H), 3.55 – 2.84 (broad m, 69H), 2.37 (s, 3H), 2.07 - 1.12 (broad m, 456H), 0.86 (t, ^{3}J = 8.1 Hz, 60H). SEC: M_n = 66 kDa, PDI = 1.85.

P8: Starting from compound **1** (124.2 mg, 0.37 mmol), compound **5** (31.9 mg, 0.41 mmol, 0.1 eq) and 2nd generation Grubbs catalyst 3.3 mg, 3.85 µmol, 0.004 eq), pure **P8** (109 mg, 69 %) was obtained as a fibrous paper like white solid using the general procedure as described for **P4**. The NMR data was identical to that of **P7**. SEC: M_n = 89 kDa, PDI = 1.92.

P9: Starting from **compound 1** (124.3 mg, 0.37 mmol), compound **5** (32.1 mg, 0.42 mmol, 0.1 eq) and 2nd generation Grubbs catalyst (3.25 mg, 3.9 µmol 0.002 eq), pure **P9** (130 mg, 82 %) was obtained as a fibrous paper like white solid using the general procedure as described for **P4**. However, in this particular case 1.5 mL of dry, degassed DCE was used and the reaction was allowed to vigorously stir for 48 h at 50 °C. The NMR data was identical to that of **P7**. SEC: M_n = 161 kDa, PDI = 1.45.

4. NMR Spectra of final monomers and polymers.

*^{1}H- and ^{13}C-NMR Spectra of final monomers **1** and **5**.*

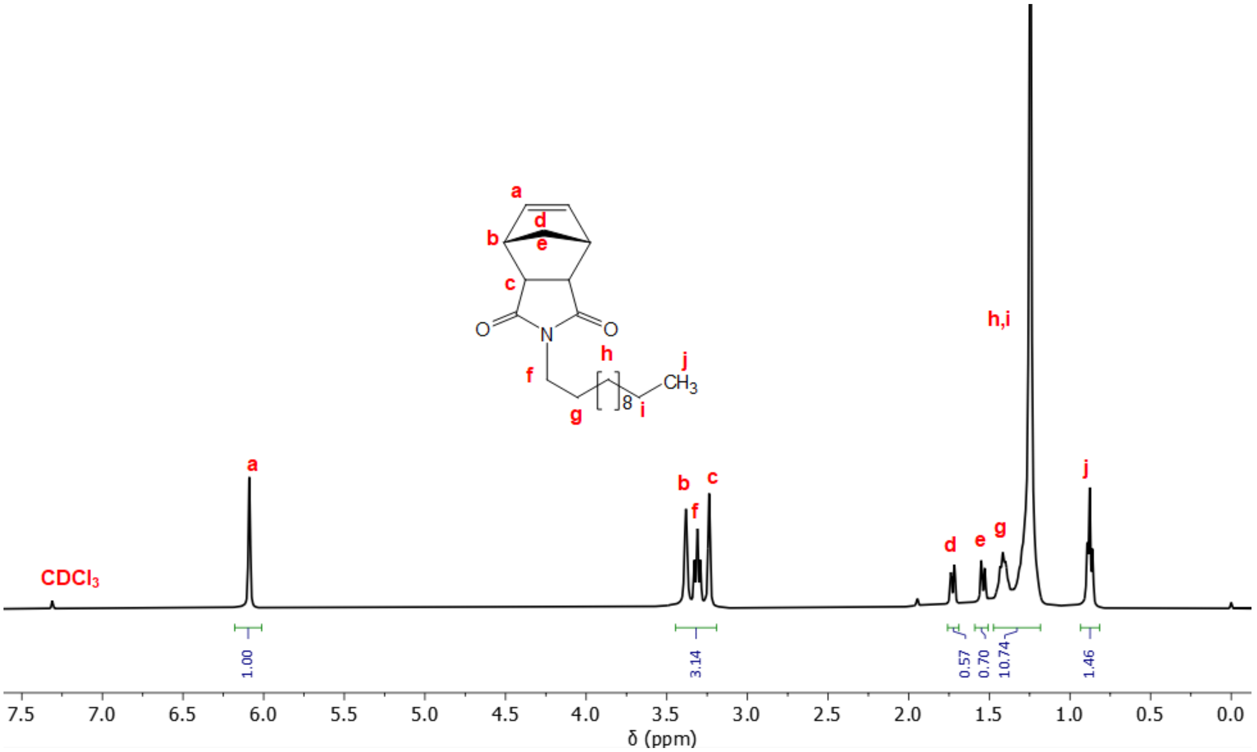

Figure S1. ^{1}H NMR spectrum of monomer **1** in chloroform-*d*.

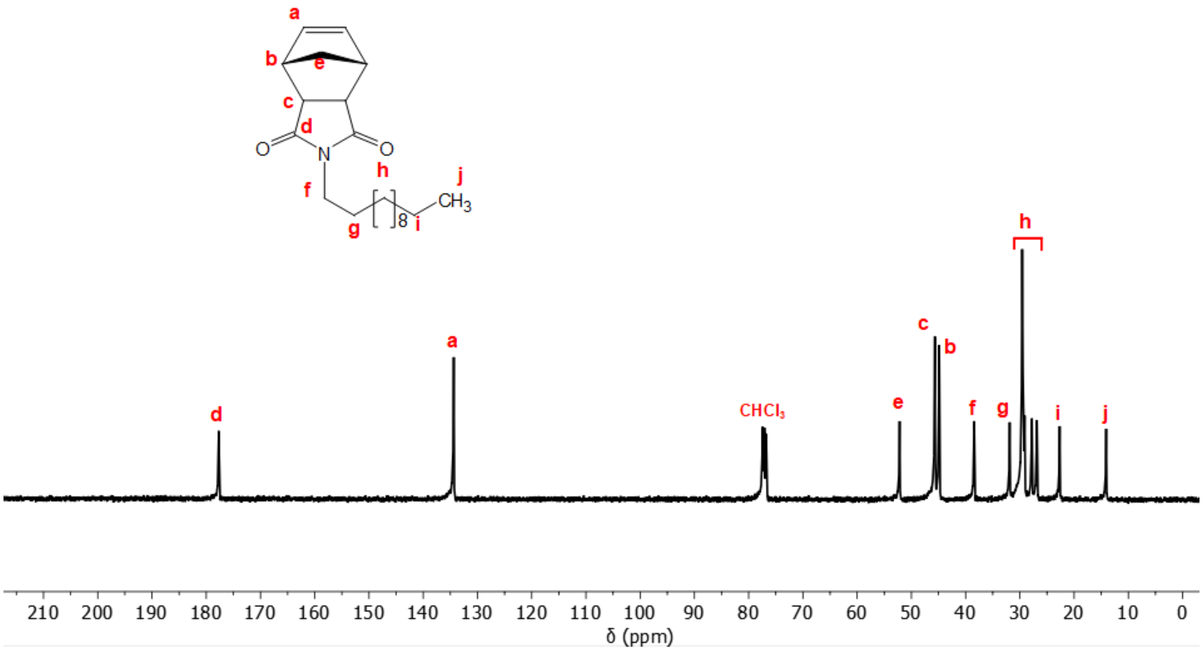

Figure S2. ^{13}C NMR spectrum of monomer **1** in chloroform-*d*.

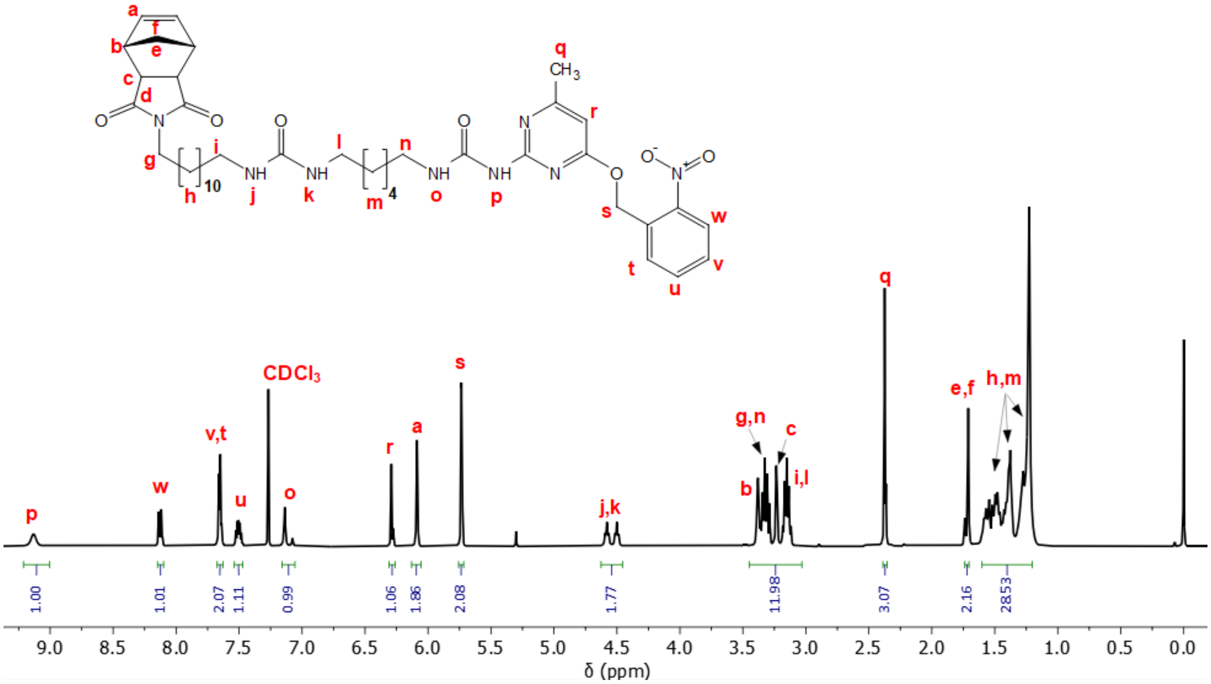

Figure S3. ^{1}H NMR spectrum of monomer **5** in chloroform-*d*.

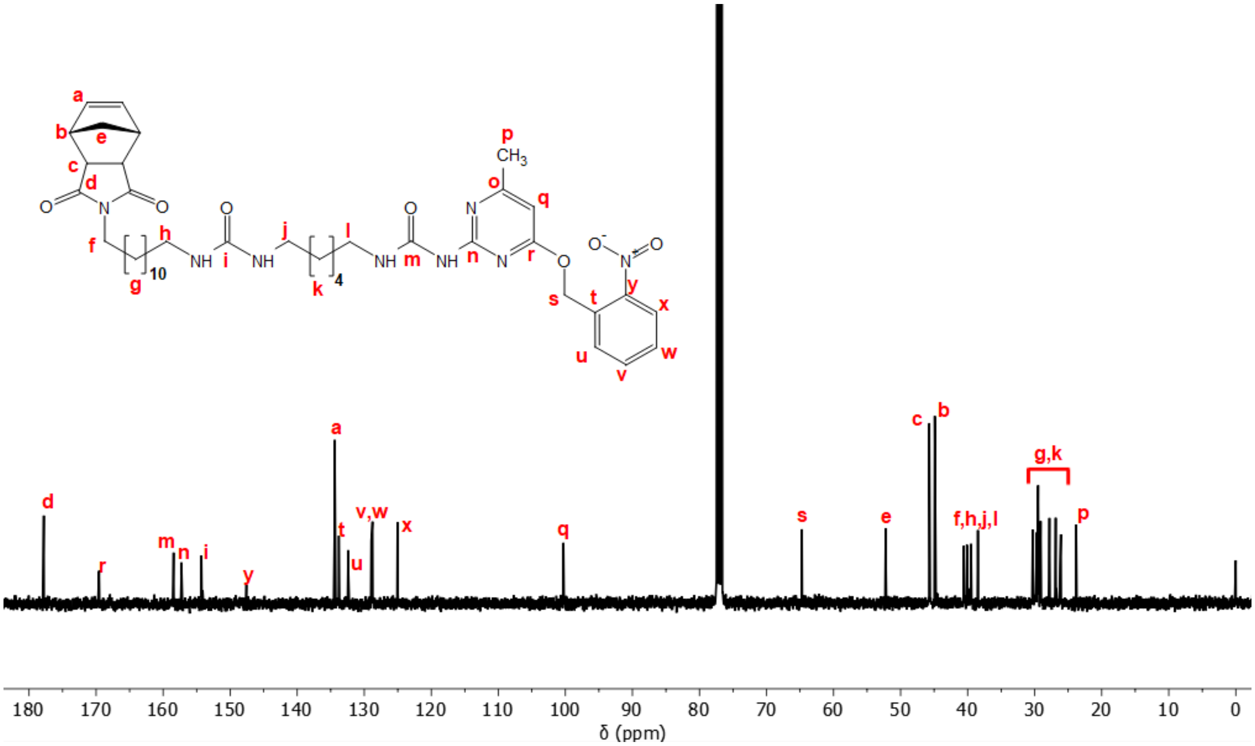

Figure S4. ^{13}C NMR spectrum of monomer **5** in chloroform-*d*.

^{1}H-NMR Spectra of non-functionalized, 5 mol% UPy and 10 mol% UPy functionalized polymers.

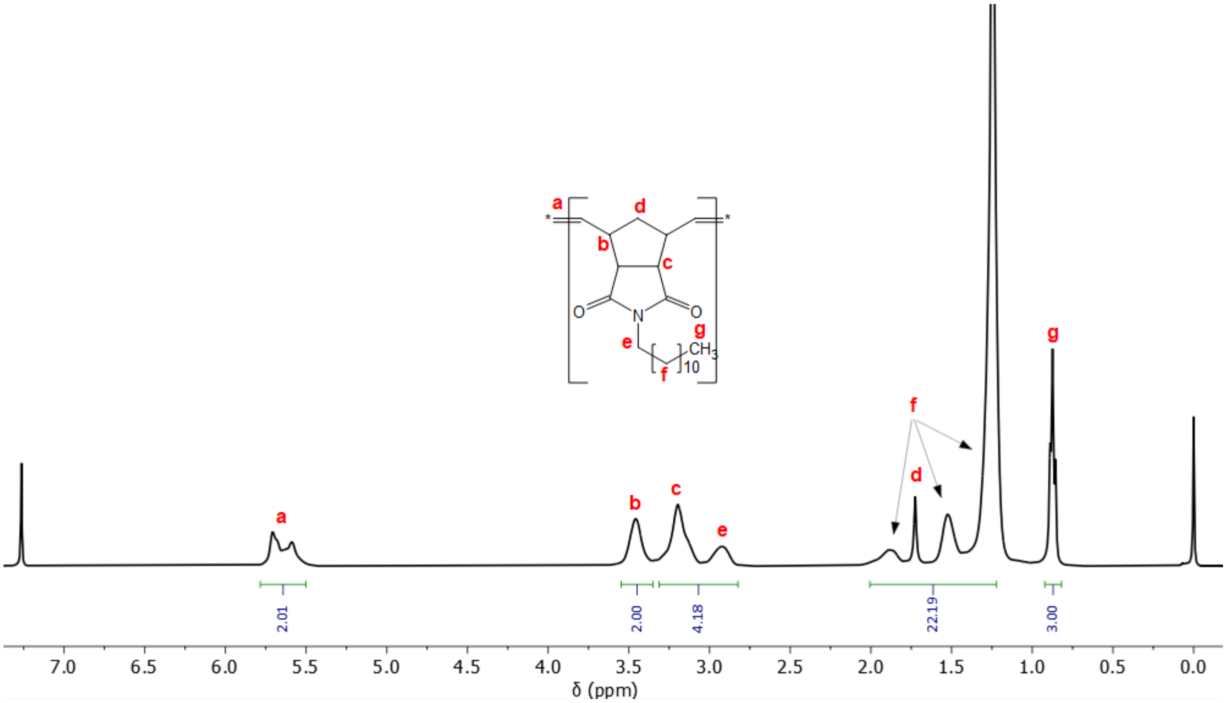

Figure S5. ^{1}H NMR spectrum of reference polymer **P2** (DP = 250) in chloroform-*d*.

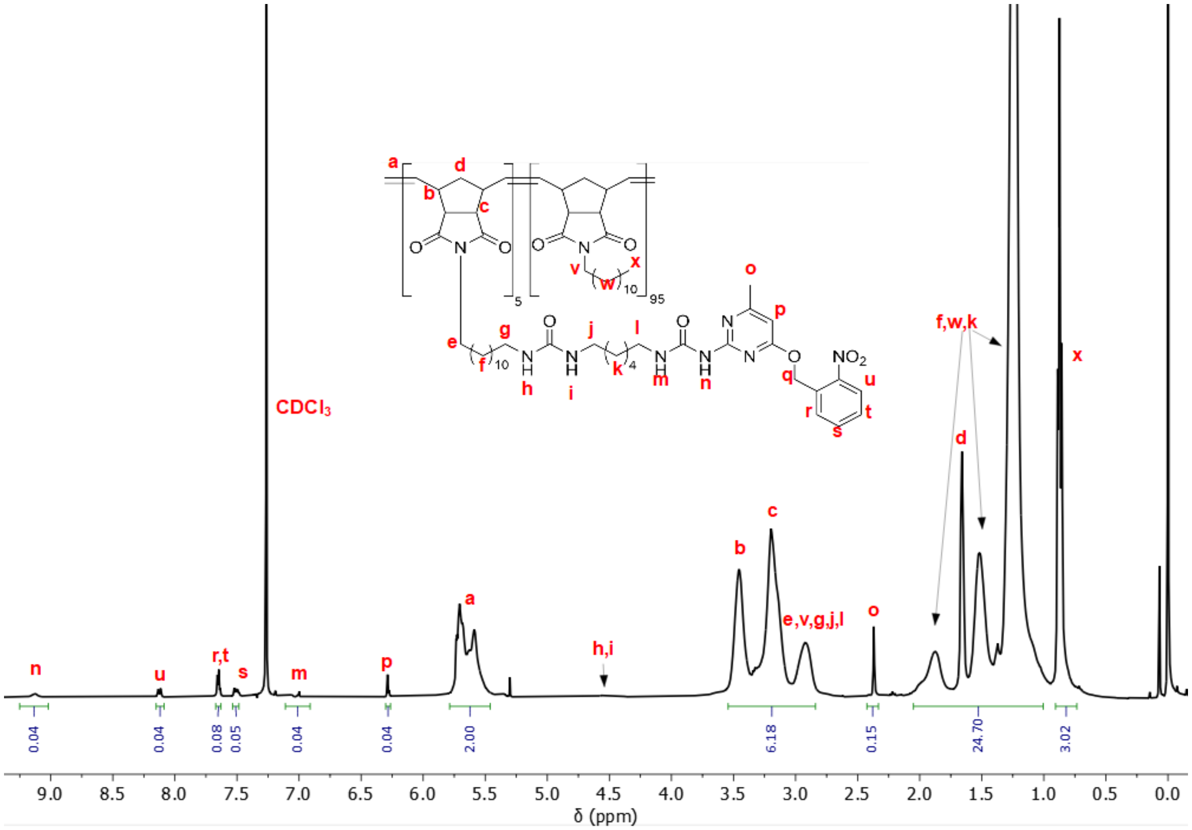

Figure S6. ^{1}H NMR spectrum of 5% functionalized polymer **P5** (DP = 250) in chloroform-*d*.

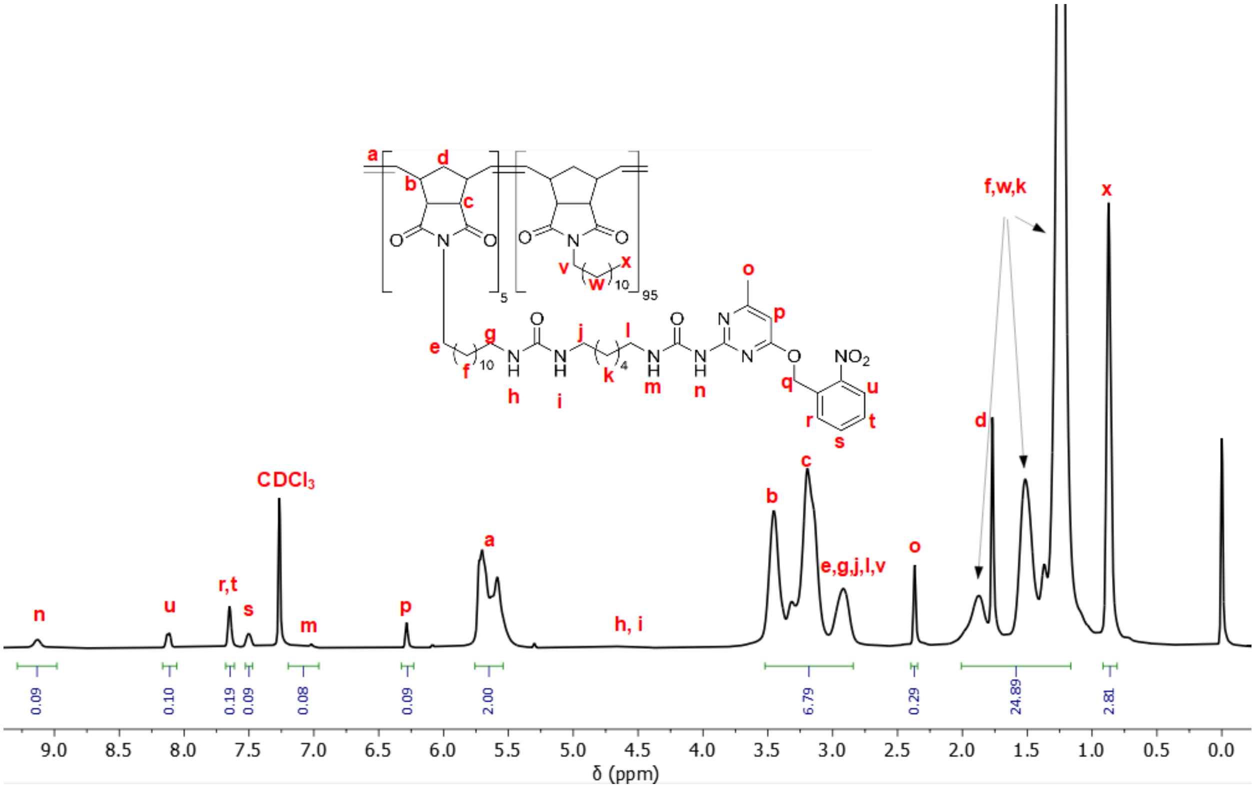

Figure S7. ^{1}H NMR spectrum of 10% functionalized polymer **P8** (DP = 250) in chloroform-*d*.

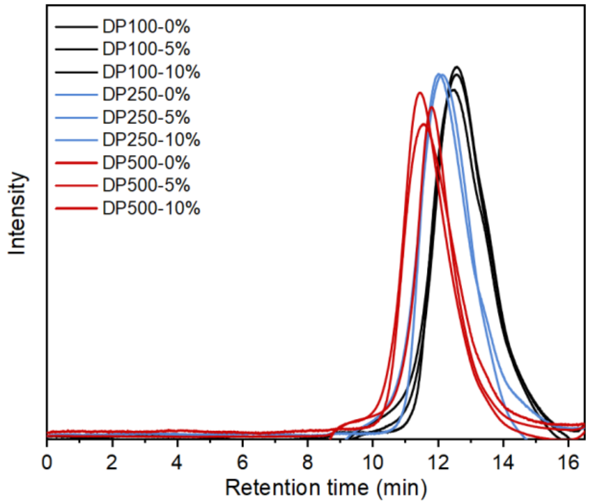

Figure S8 SEC chromatograms of polymers **P1–P9** at 1 mg mL^{-1} in THF.

5. FTIR spectra polymers P1–P9

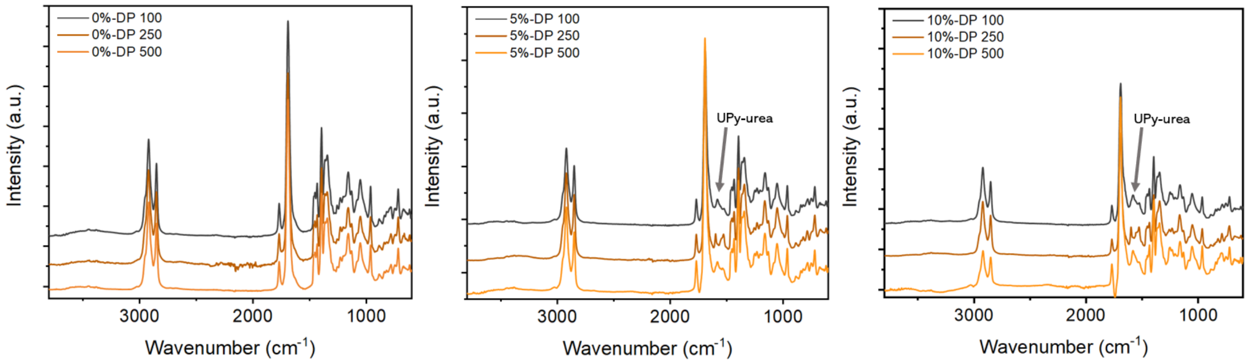

Figure S9. FTIR spectra of polymers (A) **P1–P3**, (B) **P4–P6** (5 mol% UPy-urea) and (C) **P7–P9** (10 mol% UPY-urea).

6. NMR spectra of intramolecular folding of P5

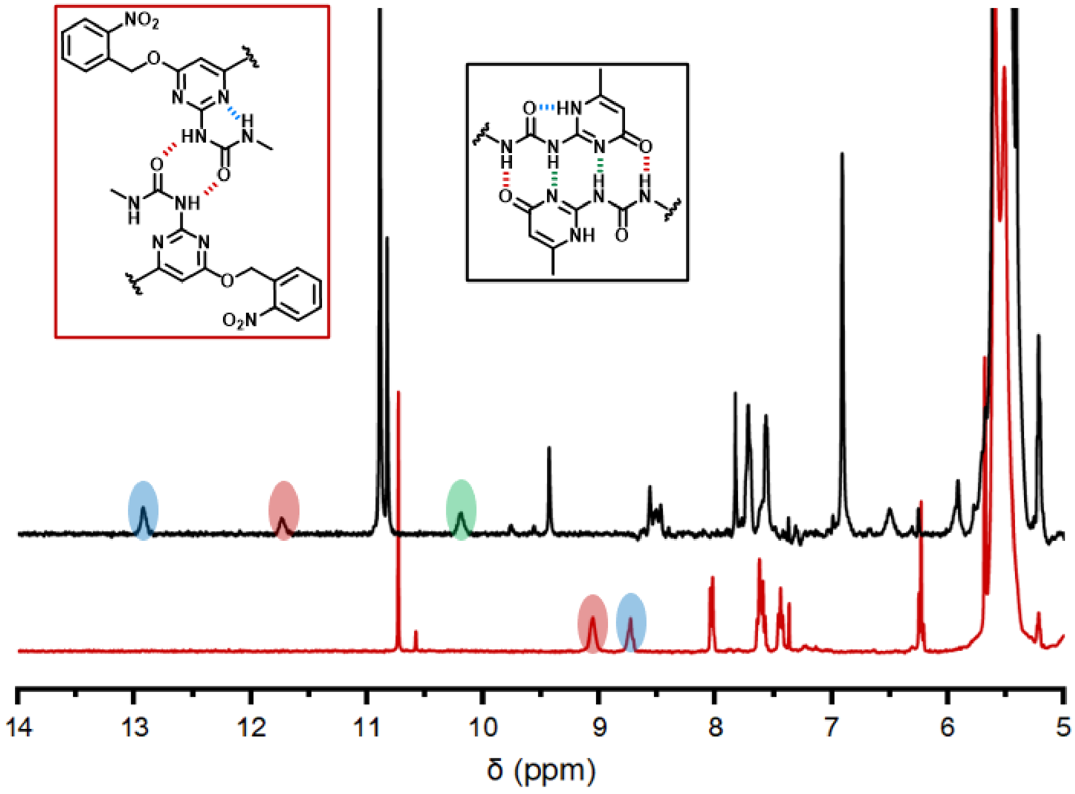

Figure S10. Folding of protected polymer **P5** (red) into intra- and intermolecularly crosslinked **P5** (black) upon UV-light deprotection (365 nm) followed by ^{1}H NMR spectroscopy.

Table S1. Polymer folding characterization of polymers **P1–P9** by SEC and DLS at mg mL^{-1} in THF.

Polymer[a]	DP[b]	UPy-urea[c] [mol%]	R_p[d] [min]	R_{dp}[e] [min]	$D_{h,p}$[f] [nm]	$D_{h,dp}$[g] [nm]	Size reduction[h] [%]
**P1**	100	0	12.5	-	15	-	-
**P2**	250	0	12.0	-	20	-	-
**P3**	500	0	11.5	-	25.5	-	-
**P4**	100	5	12.6	12.8	17	14	17.5
**P5**	250	5	12.1	12.5	24	18	25
**P6**	500	5	11.4	11.8	28.5	23.5	17.5
**P7**	100	10	12.6	12.8	21	15	29
**P8**	250	10	12.1	12.5	23	20	13
**P9**	500	10	11.8	12.1	27	23	15

[a] Polymers as depicted in Figure XX. [b] Theoretical degree of polymerization calculated from the monomer-initiator ratio used. [c] Theoretical molar percentage of UPy-urea moiety incorporated into the polymer. [d] Retention time (R_p) of protected UPy-urea grafted polymers. [e] Retention time (R_{dp}) of deprotected UPy-urea grafted polymers in their nanoparticle conformation. [f] Hydrodynamic diameter ($D_{h,p}$) of protected UPy-urea grafted polymers. [g] Hydrodynamic diameter ($D_{h,dp}$) of deprotected UPy-urea grafted polymers in their nanoparticle conformation. [h] Size reduction upon UV-deprotection determined from the obtained DLS data.

7. Thermal properties of polymer P1–P9

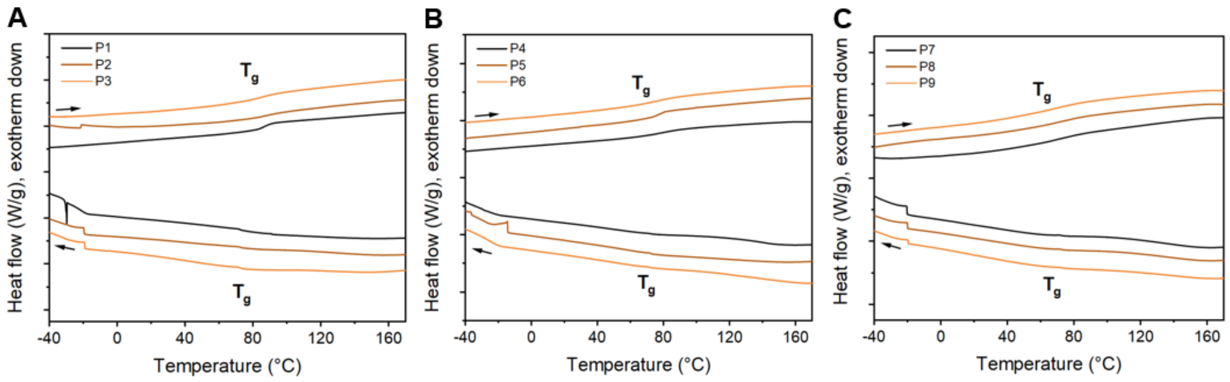

Figure S11. DSC trace of polymers (A) **P1–P3**, (B) **P4–P6** (5 mol% UPY-urea) and (C) **P7–P9** (10 mol% UPY-urea) (second heating and first cooling run). DSC traces are vertically shifted for clarity. A temperature ramp of 20 K min^{-1} was used.

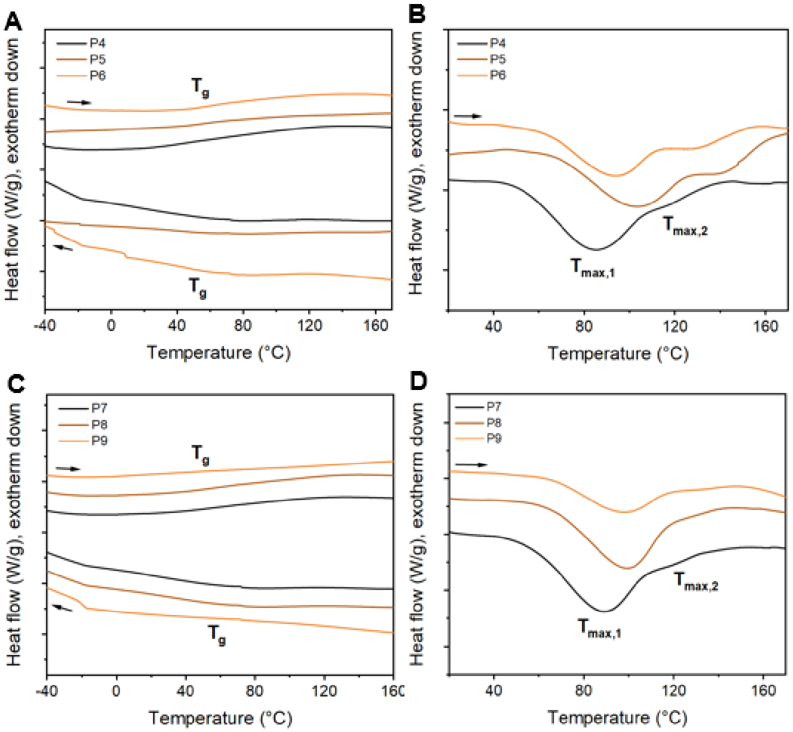

Figure S12. (A) Second heating and first cooling run and (B) corresponding first heating run of polymers **P4–P6 (intra)**. C) Second heating and first cooling run and (D) corresponding first heating run of polymers **P7–P9 (intra)**. DSC traces are vertically shifted for clarity. A temperature ramp of 10 K min^{-1} was used for the first heating run and a ramp of 20 K min^{-1} for the first cooling and second heating run.

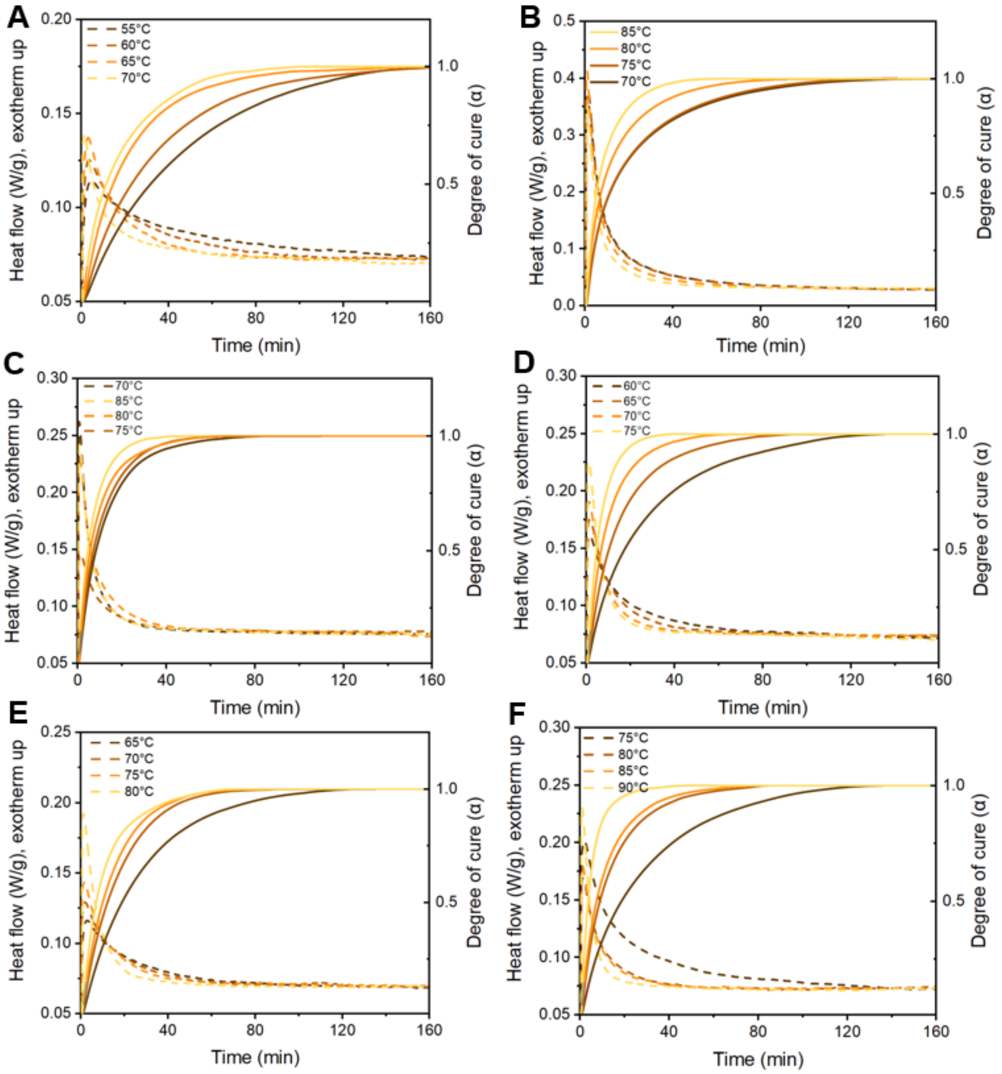

Figure S13. Isothermal DSC traces (dotted lines) and the corresponding degree of cure as a function of time (solid lines) for polymers (A) **P4 (intra)** (B), **P5 (intra)** (C) and **P6 (intra)** (D) **P7 (intra)** (E), **P8 (intra)** and (F) **P9 (intra).**

8. Redissolution of intra- and intermolecularly crosslinked polymer films of P5

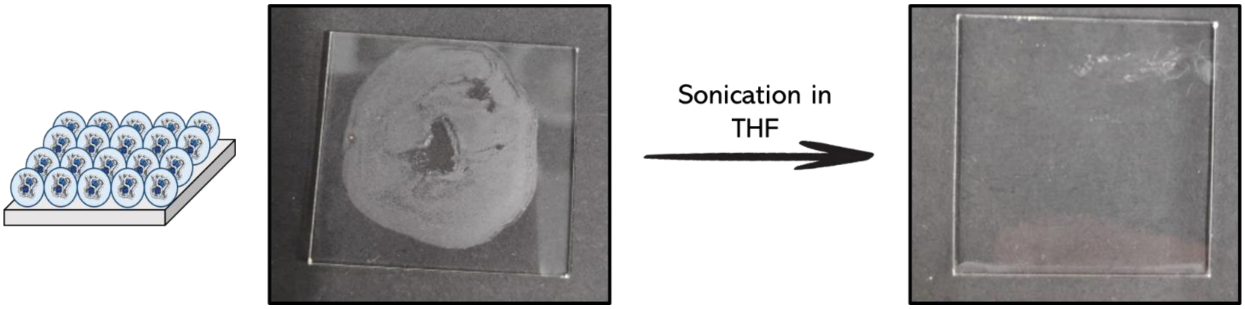

Figure S14. Visual representation of a dropcasted film of **P5 (intra)** nanoparticles on a glass slide before and after two minutes of sonication in excess THF.

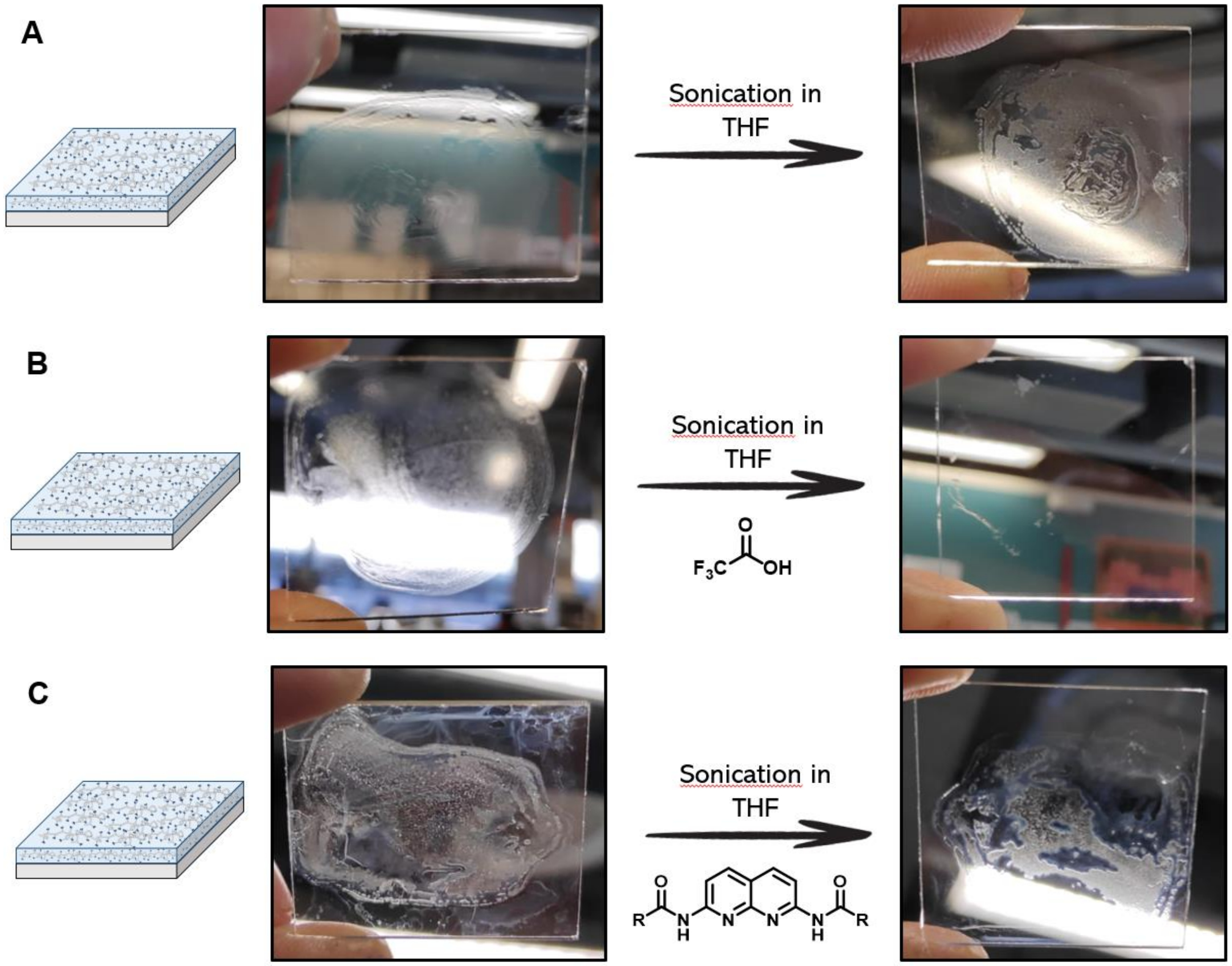

Figure S15. Visual representation of a dropcasted film of **P5 (inter)** on a glass slide before and after 2 minutes of sonication in excess THF containing (A) no additional additive, (B) TFA and (C) an excess of NaPy.
